# Evaluation of Protein Purification Techniques and Effects of Storage Duration on LC-MS/MS Analysis of Archived FFPE Human CRC Tissues

**DOI:** 10.3389/pore.2021.622855

**Published:** 2021-05-03

**Authors:** Sophia C. Rossouw, Hocine Bendou, Renette J. Blignaut, Liam Bell, Jonathan Rigby, Alan Christoffels

**Affiliations:** ^1^ South African Medical Research Council Bioinformatics Unit, South African National Bioinformatics Institute, University of the Western Cape, Bellville, South Africa; ^2^ Department of Statistics and Population Studies, University of the Western Cape, Bellville, South Africa; ^3^ Centre for Proteomic and Genomic Research, Observatory, Cape Town, South Africa; ^4^ Division of Anatomical Pathology, Department of Pathology, Faculty of Health Sciences, University of Stellenbosch, National Health Laboratory Service, Tygerberg Hospital, Cape Town, South Africa

**Keywords:** formalin-fixed paraffin-embedded proteomics, acetone precipitation and formic acid resolubilization, detergent removal plates, SP3/HILIC-on-bead-digestion, mass spectrometry, LC-MS/MS

## Abstract

To elucidate cancer pathogenesis and its mechanisms at the molecular level, the collecting and characterization of large individual patient tissue cohorts are required. Since most pathology institutes routinely preserve biopsy tissues by standardized methods of formalin fixation and paraffin embedment, these archived FFPE tissues are important collections of pathology material that include patient metadata, such as medical history and treatments. FFPE blocks can be stored under ambient conditions for decades, while retaining cellular morphology, due to modifications induced by formalin. However, the effect of long-term storage, at resource-limited institutions in developing countries, on extractable protein quantity/quality has not yet been investigated. In addition, the optimal sample preparation techniques required for accurate and reproducible results from label-free LC-MS/MS analysis across block ages remains unclear. This study investigated protein extraction efficiency of 1, 5, and 10-year old human colorectal carcinoma resection tissue and assessed three different gel-free protein purification methods for label-free LC-MS/MS analysis. A sample size of n = 17 patients per experimental group (with experiment power = 0.7 and *α* = 0.05, resulting in 70% confidence level) was selected. Data were evaluated in terms of protein concentration extracted, peptide/protein identifications, method reproducibility and efficiency, sample proteome integrity (due to storage time), as well as protein/peptide distribution according to biological processes, cellular components, and physicochemical properties. Data are available *via* ProteomeXchange with identifier PXD017198. The results indicate that the amount of protein extracted is significantly dependent on block age (*p* < 0.0001), with older blocks yielding less protein than newer blocks. Detergent removal plates were the most efficient and overall reproducible protein purification method with regard to number of peptide and protein identifications, followed by the MagReSyn^®^ SP3/HILIC method (with on-bead enzymatic digestion), and lastly the acetone precipitation and formic acid resolubilization method. Overall, the results indicate that long-term storage of FFPE tissues (as measured by methionine oxidation) does not considerably interfere with retrospective proteomic analysis (*p* > 0.1). Block age mainly affects initial protein extraction yields and does not extensively impact on subsequent label-free LC-MS/MS analysis results.

## Introduction

Tissues from biopsies, resections and/or surgery are routinely taken from patients as a treatment option and/or to facilitate more accurate diagnosis. The current universal tissue preservation method of choice is formalin-fixation and paraffin-embedment, to avoid tissue auto-proteolysis and putrefaction, and to allow tissue specimens to be analyzed and examined at a later stage [1–4]. Formalin-fixation is also considered to be a superior preservative, since formaldehyde quickly and easily penetrates and fixes tissues because of its small molecular size, it causes minimal tissue shrinkage and distortion, and produces exceptional staining results in histopathology [4–6]. The formalin-fixed, paraffin-embedded (FFPE) method of tissue preservation also allows for the indefinite room temperature storage of FFPE blocks, thereby removing much of the cost and difficulty associated with fresh-cryopreserved tissue storage. The technique involves the immersion and incubation of tissues in formaldehyde solution, which is then replaced with alcohol (ethanol) in a dehydration step. Dehydration of the sample is achieved by removing all the water from the sample *via* ethanol incubation and subsequent alcohol clearing with xylene incubation. The xylene is then replaced by molten paraffin, which infiltrates the sample. The final step involves paraffin-embedding and hardening of the sample, which involves embedment of the specimen into liquid embedding material such as wax. Samples are then stored and archived for future use [1, 3, 4].

The protein profiling of FFPE tissues has immense potential for biomarker discovery and validation. Tumor tissue represents the ideal biological material for cancer proteomics studies and biomarker discovery, since tumor-specific protein markers are typically present at elevated concentrations in patient biopsy tissue [4, 7]. Pathology institutes routinely process and store patient biopsy and/or surgery tissue samples and therefore most pathology archives consist of thousands of FFPE blocks, which often comprise recent as well as decade-old blocks. These repositories contain numerous varieties of patient tissue specimens, including rare malignancies together with metadata such as patient medical records, which contain information about diagnosis, survival, and response to therapy. Due to this and the fact that FFPE samples are easily stored and obtainable, many recent proteomics, genomics and immunohistochemical studies have focused on improving methods for analysis of FFPE tissue [4, 8, 9]. However, the effect of long-term storage, at resource-limited institutions in developing countries, on extractable protein quantity/quality has not yet been investigated. In addition, the optimal sample preparation techniques required for accurate, reproducible results from label-free LC-MS/MS analysis across block ages remains unclear.

[10] found no significant difference in protein identifications from FFPE kidney tissue (normal and tumor) samples that were stored up to 10 years. In addition, some top-down proteomic studies have found no significant difference in protein yields between younger and older FFPE blocks [11], whereas others have found a significant decrease in protein yield as block age increases [12, 13]. The main detrimental pre-analytical factor appears to be tissue fixation time, with longer periods (>24 h) leading to significant decreases in protein yield and number of proteins identified *via* LC-MS/MS [13–15]. During the completion of this study [16], published their work in which they used tandem mass tag labeling and high pH fractionation to evaluate the impact of storage time on FFPE ovarian adenocarcinoma specimens (as old as 32 years) and found an overall decline in identifiable peptides and phosphopeptides due to the formalin fixation process but no further decline/degradation due to storage duration. Even though the aforementioned studies focused on storage duration/block age, to our knowledge there is no evidence to demonstrate the outcome of different protein purification techniques on older samples. There remains a need to provide empirical evidence for the impact of storage duration and conditions within the context of a resource-limited environment, such as the Anatomical Pathology department at Tygerberg Hospital (Western Cape, South Africa).

Due to formalin-induced protein cross-linking, strong detergents such as sodium dodecyl sulfate (SDS) are required for total tissue solubilization and protein extraction from FFPE tissues [17–19]. However, SDS binds to amino acids and thereby changes the protein spatial conformational structure. This, in turn, inhibits proteases, such as trypsin, from accessing protein cleavage sites (which have become distorted through SDS binding) and also inhibits protease activity by changing enzyme conformational structure (through SDS binding). In addition, SDS alters the chromatographic separation of peptides and also interferes with electrospray ionization (ESI) mass spectrometry by dominating mass spectra and significantly suppressing analyte ion signals since it is readily ionizable and present in greater abundances than individual peptide ions. For these reasons, SDS must be completely depleted from a sample before enzymatic digestion and LC-ESI MS/MS analysis [17–20]. However, SDS removal with minimal sample loss is a challenging task and several gel-free approaches have been proposed over the years. These approaches include incorporating the use of detergent removal plates (DRP), protein precipitation with organic solvents, such as the acetone precipitation and formic acid resolubilization (APFAR) method [18, 20, 21], and/or methods using hydrophilic interaction liquid chromatography (HILIC) and magnetic resin (such as the Single-Pot Solid-Phase-enhanced Sample Preparation (SP3) method) [22] in the sample processing workflow prior to LC-MS/MS analysis.

One of the aims of this study was to methodically characterize the effects of storage time (over 1, 5, and 10 years) on the quality of data produced *via* label-free LC-MS/MS analysis of FFPE tissue blocks from a resource-limited pathology archive, to dispel any notions that these samples may be inferior for whatever reason so that they can be utilized with confidence in any future studies. In addition, three different gel-free protein purification methods (APFAR, DRP and MagReSyn^®^ SP3/HILIC) for label-free LC-MS/MS analysis were also assessed across all block ages. These protein purification methods were published within the last 5 years, and their comparative analysis have not been carried out to our knowledge and this study provides experimental data for this assessment together with statistical support. Furthermore, the best suited method for analyzing archived colorectal carcinoma (CRC) FFPE tissue was determined with regards to peptide and protein identifications, reproducibility, digestion efficiency, and any method-based protein selection bias.

## Materials and Methods

### FFPE Human Colorectal Carcinoma Resection Samples

FFPE tissue blocks, which consist of human CRC resection samples, were obtained from the Anatomical Pathology department at Tygerberg Hospital (Western Cape, South Africa) after obtaining ethics approval from the Biomedical Science Research Ethics Committee (BMREC) of the University of the Western Cape, as well as the Health Research Ethics Committee (HREC) of Stellenbosch University. The FFPE blocks were anonymized prior to processing. The 1-year-old blocks were archived since approximately 2016 (when the tissue was resected), 5-year-old blocks were archived since 2012, and 10-year-old blocks were archived since 2007 (experiments/protein extractions were performed in 2017/2018). Tissue processing and fixation times/conditions and storage conditions are unknown, since specimens were retrospectively collected. Seventeen patient cases, per block age, were reviewed and selected (Table 1). Using protein identification results from a pilot study (unpublished results), an overall F-test for one-way ANOVA determined that the sample size (*n* = 17) per group/block age resulted in a calculated power of 0.7 (*α* = 0.05).

**TABLE 1:** Information of the FFPE specimens selected for analysis.

Patient number	Block age (years)	Patient age (years)	Gender	Diagnosis	Grade	Stage	Location
1	1	75	M	Adenocarcinoma	Low-grade	IIA	Left colon
2	1	81	M	Adenocarcinoma	Low-grade	IIA	Left colon
3	1	68	F	Adenocarcinoma	Low-grade	IIA	Left colon
4	1	42	M	Adenocarcinoma	Low-grade	IVA	Left colon
5	1	80	F	Adenocarcinoma	Low-grade	I	Left colon
6	1	79	M	Adenocarcinoma	Low-grade	IIA	Left colon
7	1	49	M	Adenocarcinoma	Low-grade	IIA	Left colon
8	1	40	F	Adenocarcinoma	Low-grade	IIA	Left colon
9	1	56	M	Adenocarcinoma	Low-grade	IIA	Left colon
10	1	79	F	Adenocarcinoma	Low-grade	IIA	Left colon
11	1	64	F	Adenocarcinoma	Low-grade	IIA	Left colon
12	1	53	M	Adenocarcinoma	Low-grade	IIIB	Left colon
13	1	78	M	Adenocarcinoma	Low-grade	IIA	Left colon
14	1	51	F	Adenocarcinoma	Low-grade	IIIB	Left colon
15	1	31	M	Adenocarcinoma	Low-grade	IIIB	Left colon
16	1	73	F	Adenocarcinoma	Low-grade	IIIB	Left colon
17	1	54	F	Adenocarcinoma	Low-grade	IIIC	Left colon
18	5	51	F	Adenocarcinoma	Low-grade	IIA	Left colon
19	5	56	F	Adenocarcinoma	Low-grade	IIIB	Left colon
20	5	86	M	Adenocarcinoma	Low-grade	IIA	Left colon
21	5	59	M	Adenocarcinoma	Low-grade	IIC	Left colon
22	5	67	M	Adenocarcinoma	Low-grade	IIA	Left colon
23	5	82	M	Adenocarcinoma	Low-grade	IIA	Left colon
24	5	49	F	Adenocarcinoma	Low-grade	IIIB	Left colon
25	5	54	M	Adenocarcinoma	Low-grade	IIA	Left colon
26	5	58	M	Adenocarcinoma	Low-grade	IIC	Left colon
27	5	44	F	Adenocarcinoma	Low-grade	I	Left colon
28	5	50	M	Adenocarcinoma	Low-grade	IIA	Left colon
29	5	74	F	Adenocarcinoma	Low-grade	IIA	Left colon
30	5	54	M	Adenocarcinoma	Low-grade	IIA	Left colon
31	5	47	F	Adenocarcinoma	Low-grade	IIIA	Left colon
32	5	55	M	Adenocarcinoma	Low-grade	IIIB	Left colon
33	5	83	M	Adenocarcinoma	Low-grade	IIA	Left colon
34	5	60	M	Adenocarcinoma	Low-grade	IIA	Left colon
35	10	69	M	Adenocarcinoma	Low-grade	IIIB	Left colon
36	10	47	F	Adenocarcinoma	Low-grade	IIA	Left colon
37	10	58	F	Adenocarcinoma	Low-grade	IIA	Left colon
38	10	83	M	Adenocarcinoma	Low-grade	IIA	Left colon
39	10	57	F	Adenocarcinoma	High-grade	IIA	Right colon
40	10	46	F	Adenocarcinoma	High-grade	IIA	Right colon
41	10	77	F	Adenocarcinoma	Low-grade	IIA	Left colon
42	10	63	F	Adenocarcinoma	Low-grade	IIA	Left colon
43	10	67	M	Adenocarcinoma	Low-grade	IIIB	Left colon
44	10	50	F	Adenocarcinoma	Low-grade	IIA	Left colon
45	10	42	M	Adenocarcinoma	Low-grade	IIA	Left colon
46	10	71	F	Adenocarcinoma	Low-grade	IIA	Left colon
47	10	70	M	Adenocarcinoma	Low-grade	IIA	Left colon
48	10	69	M	Adenocarcinoma	Low-grade	IIA	Left colon
49	10	62	F	Adenocarcinoma	Low-grade	IIA	Right colon
50	10	78	M	Adenocarcinoma	Low-grade	IIIB	Left colon
51	10	33	M	Adenocarcinoma	Low-grade	IIA	Left colon

**FIGURE 1:**
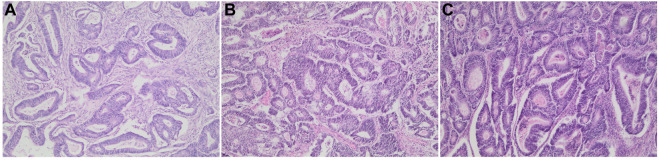
Colonic adenocarcinoma resection tissue samples. Representative H&E stained sections of patient cases/block ages analyzed in this study. **(A)** 1-year-old block. **(B)** 5-year-old block, and **(C)** 10-year-old block at ×100 magnification.

Patients diagnosed with colorectal adenocarcinoma, after H&E staining, were reviewed by a pathologist to ensure tissue quality and comparability (Figure 1). The selected slides had carcinomas with more than 90% viable tumor nuclei.

### Protein Extraction and Quantification

**FIGURE 2:**
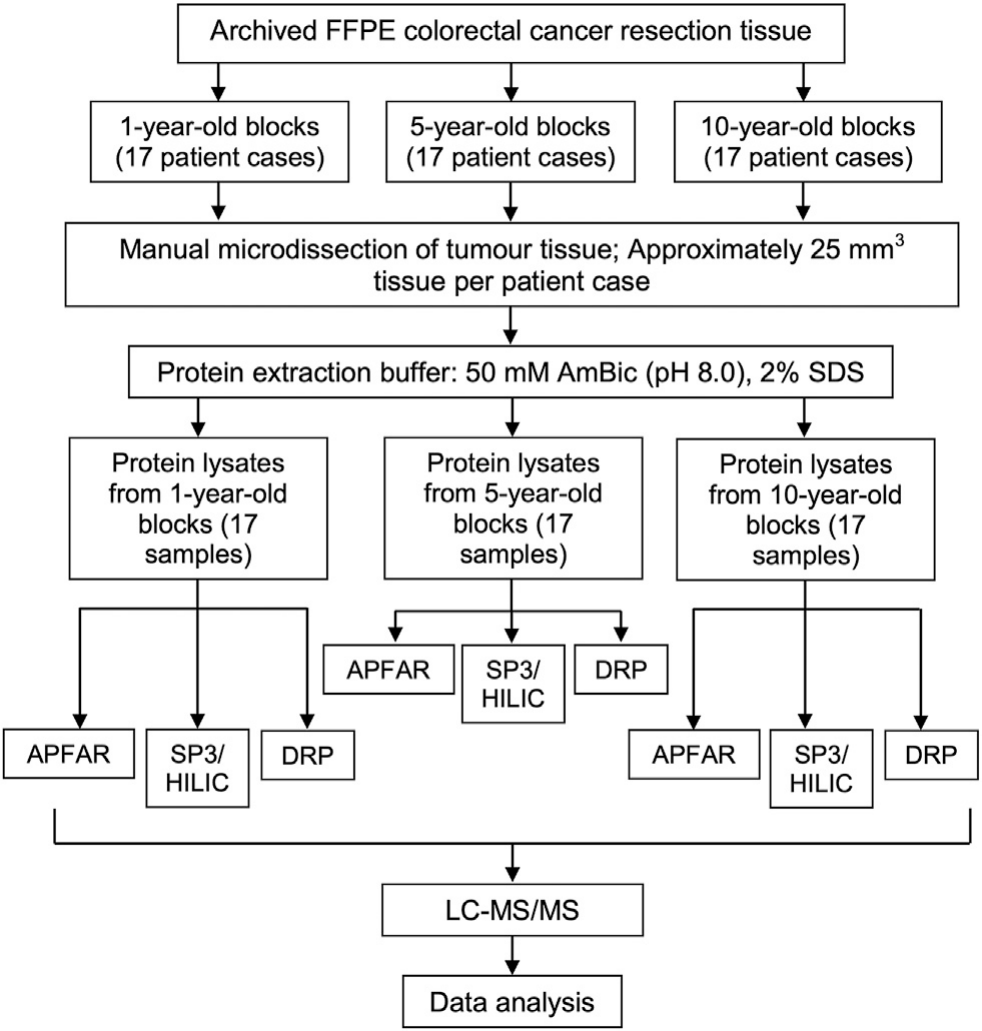
Experimental design and workflow used to evaluate the effects of block age and different sample processing methods. FFPE human colorectal carcinoma resection tissues from 17 patients per block age (1, 5, and 10-year old blocks) were cut and tumor areas were manually micro-dissected for analysis. From each patient, tissue sections, which corresponded to approximately 25 mm^3^ tissue per patient/sample, were cut per sample. Protein was extracted and quantified, after which each patient sample was split in three, for subsequent sample processing by either the APFAR, DRP, or SP3/HILIC methods. Resultant peptides were analyzed *via* LC-MS/MS and data analysis was performed on all sample MS/MS spectra.

For each selected patient case (*n* = 17 per experimental condition), a number of 25 µm sections, which were equivalent to 25 mm^3^ of manually micro-dissected FFPE tumor tissue per sample, were cut and mounted onto generic glass microscopy slides. Sections were air dried and processed for protein extraction as is shown in Figure 2 (five batches of samples, with randomized selection and inclusion of samples from each of the different storage times, were processed for protein extraction.) The method used for sample processing and protein extraction was modified from the protocols used by [23, 24]. Briefly, tissue sections (mounted on glass slides) were heated on a heating block (65°C for 5 min), to melt the paraffin wax, followed by tissue deparaffinization consisting of two consecutive incubations in xylene (Sigma-Aldrich, United States) for 2.5 min and 1.5 min each respectively, at room temperature. Tissue sections were then rehydrated by successive incubations in absolute ethanol (Merck, Germany), 70% (v/v) ethanol, and twice with distilled water, for 1 min each at room temperature. The tissues were collected in protein LoBind microcentrifuge tubes (Eppendorf, Germany) by scraping the tissue off the glass slides using a clean sterile scalpel blade. Protein extraction buffer (50 mm Ammonium bicarbonate (AmBic) (pH 8.0) (Sigma-Aldrich, United States), 2% (w/v) SDS (Sigma-Aldrich, United States) was added to the samples at a volume of approximately 20 µl protein extraction buffer per mm^3^ of tissue (approximately 25 mm^3^ tissue per sample). Samples were mixed by vortexing and incubated at 99°C in a heating block with agitation set at 600 RPM for 1 h, after which the samples were cooled/placed on ice before centrifugation at 16,000 x g and 18°C for 20 min to pellet the cell debris. The clarified lysates of each sample was transferred to new protein LoBind microcentrifuge tubes and an aliquot taken for protein yield determination. All samples were stored at −80°C until further processing. For protein yield determination, the total protein extracted from the FFPE tissues were quantified using the Pierce™ BCA Protein Assay Kit (Pierce Biotechnology, Thermo Fisher Scientific, United States) according to manufacturer’s instructions. The samples were subsequently processed by the DRP, APFAR [18, 20, 21] and/or MagReSyn^®^ SP3/HILIC magnetic bead digestion method [25]; ReSyn Biosciences, South Africa), prior to LC-MS/MS analysis (Figure 2).

### Protein Purification Methods

#### Detergent Removal Plates Method

Detergent removal was carried out using detergent removal spin plates (Pierce Biotechnology, Thermo Fisher Scientific, United States) according to the manufacturer’s instructions. Briefly, a detergent removal plate was placed on top of a wash plate and the shipping solution spun out at 1,000 x g for 2 min. The resin bed was equilibrated with 300 µl of 50 mm Triethylammonium bicarbonate (TEAB) and spun through as before, and this was repeated twice. Thereafter, 100 µg of protein was loaded onto the columns and incubated at room temperature for 2 min before spinning through at 1,000 x g for 2 min into the sample collection plate. Samples were then transferred to protein Lobind tubes and dried down by vacuum centrifugation. Once dried, samples were resuspended in 30 µl of 50 mm TEAB.

#### Acetone Precipitation and Formic Acid Resolubilization Method

A total of 100 µg protein was transferred to each protein Lobind microcentrifuge tube and precipitated by addition of four volumes of ice cold acetone (Sigma-Aldrich, United States) followed by overnight incubation at −20°C. Samples were then centrifuged at 21,000 x g for 15 min at 4°C. The supernatant was discarded and the pellet washed with ice cold acetone. This process was repeated for a total of three pelleting steps. Thereafter, the pellets were air-dried and subsequently solubilized by resuspension in 50 mm TEAB.

#### In-Solution Digestion

In-solution digestion was carried out on samples processed by the APFAR and DRP methods. The protein was reduced by the addition of 0.1 volumes of 100 mm tris(2-carboxyethyl)phosphine (TCEP) (Sigma-Aldrich, United States) to each sample followed by incubation at 60°C for 1 h. Alkylation was accomplished by addition of 0.1 volumes of 100 mm methyl methanethiosulphonate (MMTS) (Sigma-Aldrich, United States), which was prepared in isopropanol (Sigma-Aldrich, United States), to each sample and subsequent incubation at room temperature for 15 min. Protein digestion was accomplished by addition of 1:50 (trypsin: final protein ratio) trypsin (Promega, United States) in a solution with 50 mm TEAB, and overnight incubation at 37°C. Samples were dried down and resuspended in 0.1% trifluoroacetic acid (TFA) (Sigma-Aldrich, United States) prior to clean-up *via* Zip-Tip (Sigma-Aldrich, United States), after which the samples were again dried down and resuspended in a final volume of 12 µl liquid chromatography (LC) loading buffer (0.1% formic acid (FA) (Sigma-Aldrich, United States), 2% Acetonitrile (ACN) (Burdick and Jackson, United States).

#### MagReSyn^®^ SP3/HILIC Method With On-Bead Digestion

In preparation for the SP3/HILIC magnetic bead workflow, MagReSyn^®^ HILIC beads (ReSyn Biosciences, South Africa) were aliquoted into a new tube and the shipping solution removed. Beads were then washed with 250 µl wash buffer (15% ACN, 100 mm Ammonium acetate (Sigma-Aldrich, United States) pH 4.5) for 1 min then resuspended in loading buffer (30% ACN, 200 mm Ammonium acetate, pH 4.5). The rest of the process, described hereafter, was performed using a Hamilton MassSTAR robotics liquid handler (Hamilton, Switzerland). A total of 50 µg of protein from each sample was transferred to a protein LoBind plate (Merck, Germany). Protein was reduced with 10 mm TCEP (Sigma-Aldrich, United States) and incubated at 60°C for 1 h. Samples were cooled to room temperature and alkylated with 10 mm MMTS (Sigma-Aldrich, United States) at room temperature for 15 min. MagReSyn^®^ HILIC magnetic beads were added at an equal volume to that of the sample and a ratio of 5:1 total protein. The plate was incubated at room temperature on a shaker at 900 RPM for 30 min for binding of protein to beads. After binding, the beads were washed four times with 500 µl of 95% ACN for 1 min each. For digestion, trypsin (Promega, United States) made up in 50 mm TEAB was added at a ratio of 1:10 total protein, and the plate was incubated at 37°C on the shaker for 4 h. After digestion, the supernatant containing the peptides was removed and dried down. The samples were then resuspended in LC loading buffer [0.1% FA (Sigma-Aldrich, United States), 2% ACN (Burdick & Jackson, United States)].

### Label–Free LC–MS/MS Analysis

LC-MS/MS analysis was conducted with a Q-Exactive quadrupole-Orbitrap mass spectrometer (Thermo Fisher Scientific, United States) coupled with a Dionex Ultimate 3,000 nano-UPLC system. All samples run by LC-MS/MS were in a randomized order. Peptides were dissolved in a solution of 0.1% FA and 2% ACN and loaded on a C18 trap column (PepMap100, 300 µm × 5 mm × 5 µm). Samples were trapped onto the column and washed for 3 min before the valve was switched and peptides eluted onto the analytical column as described hereafter. A gradient of increasing organic proportion was used for peptide separation - chromatographic separation was performed with a Waters nanoease (Zenfit) M/Z Peptide CSH C18 column (75 µm × 25 cm × 1.7 µm) and the solvent system employed was solvent A [0.1% FA in LC water (Burdick and Jackson, United States)] and solvent B (0.1% FA in ACN). The multi-step gradient for peptide separation was generated at 300 nl/min as follows: time change 5 min, gradient change: 2–5% solvent B, time change 40 min, gradient change 5–18% solvent B, time change 10 min, gradient change 18–30% solvent B, time change 2 min, gradient change 30–80% solvent B. The gradient was then held at 80% solvent B for 10 min before returning it to 2% solvent B and conditioning the column for 15 min. All data acquisition was obtained using Proxeon stainless steel emitters (Thermo Fisher, United States). The mass spectrometer was operated in positive ion mode with a capillary temperature of 320°C. The applied electrospray voltage was 1.95 kV. The mass spectra were acquired in a data-dependent manner using Xcalibur™ software version 4.2 (Thermo Fisher, United States) (Details of data acquisition parameters are shown in Supplementary Table S1).

### Peptide and Protein Identification

Raw data containing centroid MS/MS spectra were converted into mgf (Matrix Science, United Kingdom) files using msconvert from the Proteo-Wizard software suite [12]. Peak lists obtained from MS/MS spectra were identified using X!Tandem (version X!Tandem Vengeance 2015.12.15.2) [26], MS Amanda (version 2.0.0.9706) [27] and MS-GF+ (version 2018.04.09) [28]. The search was conducted using SearchGUI (version 3.3.3) [29]. Protein identification was conducted against a concatenated target/decoy [30] version of the *Homo sapiens* (20,341, >99.9%) [with *Sus scrofa* (1, <0.1%)] complement of the UniProtKB [31] human reviewed Swiss-Prot proteome (one trypsin *Sus scrofa* sequence was also obtained from UniProtKB), downloaded on May 21, 2018 (Supplementary Data Sheet S1). The decoy sequences were created by reversing the target sequences in SearchGUI. The identification settings were as follows: Trypsin, Specific, with a maximum of 2 missed cleavages; 10.0 ppm as MS1 and 0.02 Da as MS2 tolerances; fixed modifications: Methylthio of C (+45.987,721 Da), variable modifications: Oxidation of M (+15.994,915 Da), Deamidation of N and Q (+0.984,016 Da); fixed modifications during refinement procedure: Methylthio of C (+45.987,721 Da), variable modifications during refinement procedure: Acetylation of protein N-term (+42.010565 Da), Pyrolidone from E (--18.010565 Da), Pyrolidone from Q (--17.026549 Da), Pyrolidone from carbamidomethylated C (--17.026549 Da). All algorithms specific settings are listed in the certificate of analysis available in Supplementary Data Sheet S1. Peptides and proteins were inferred from the spectrum identification results using PeptideShaker version 1.16.40 [32]. Peptide Spectrum Matches (PSMs), peptides and proteins were validated at a 1% False Discovery Rate (FDR) estimated using the decoy hit distribution (example of an annotated MS/MS spectrum for a peptide is shown in Supplementary Image S1). All validation thresholds are listed in the certificate of analysis (Supplementary Data Sheet S1). Post-translational modification localizations were scored using the D-score [33] and the phosphoRS score [34] with a threshold of 95.0 as implemented in the compomics-utilities package [35] (example of post-translational modification localizations for a peptide is shown in Supplementary Image S2).

### Data and Statistical Analyses

Qualitative and quantitative data were exported from PeptideShaker and parsed using in-house scripts and graphs generated in Jupyter lab (using Pandas, NumPy, and Matplotlib Python packages), as well as Microsoft^®^ Excel. Additional statistical analyses were performed using SAS^®^ university edition and SAS^®^ Studio version 3.8 (results of the statistical tests that were performed are listed in Supplementary Table S2). To determine if sample distributions were normal, a Kolmogorov–Smirnov or Shapiro–Wilk test was performed, with D denoting the test statistic for the Kolmogorov–Smirnov test and W denoting the test statistic for the Shapiro–Wilk test. For normal distributions, comparison of means across 3 (or more) groups was performed using the parametric ANOVA procedure, with F (F-ratio) denoting the test statistic. The Kruskal–Wallis nonparametric test for medians was used when data were from a non-normal distribution, and H denotes the test statistic. For all statistical reporting, the test statistic value is given along with the degrees of freedom (in brackets after the test statistic symbol) and *p*-value. Post hoc statistical analyses were performed on significant results using Bonferroni or Dunn’s test [36]; Elliott and Hynan, 2011).

Spectrum counting abundance indexes were estimated using the Normalized Spectrum Abundance Factor (NSAF) [37] adapted for better handling of protein inference issues and peptide detectability. The NSAF method followed here involves counting the number of spectra attributed to each protein in the result set, which is subsequently normalized to a relative abundance [29, 37, 38]. In the PeptideShaker implementation, this count is then normalized for the length of the protein, the presence of shared peptides, as well as redundant peptides [29, 38]. The spectrum counting indexes were exported from PeptideShaker and parsed using in-house scripts. The NSAF values were multiplied by the lowest factor calculated for each pair of conditions compared, in order to deal with integers and facilitate comparisons. These NSAF values were then used to estimate the extent of differential protein abundance by calculating the Pearson’s correlation coefficient (PCC), for each pair of conditions compared, to assess the relationship/level of correlation between samples. PCC graphs were generated in Jupyter lab using Pandas, NumPy, and Matplotlib Python packages. Principal component analysis (PCA) was performed for each patient case/sample’s list of identified proteins and corresponding NSAF values, with Jupyter lab, using Pandas, NumPy, Scikit-learn, Seaborn and Matplotlib Python packages.

The physicochemical properties of the identified peptides, including the hydropathicity (Kyte-Doolittle scale), molecular weight, and isoelectric point were calculated for each sample using the Protein property analysis software (ProPAS) version 1.1 [39].

Venny version 2.1.0 [40] was used to generate Venn diagrams to visualize the consistency of peptide identifications between samples.

Protein annotations regarding subcellular localization were retrieved from Ensembl^1^ using GOSlim UniProtKB-GOA^2^ to minimize the number of terms retrieved. Hypergeometric testing was used to calculate the significance of gene ontology terms.

Inkscape Version 0.92.4 (5da689c313, 2019–01-14) (https://www.inkscape.org) was used to combine multiple graphs into single figures, add color and/or patterns and increase figure resolution.

### Data Sharing Information

The mass spectrometry proteomics data [41] have been deposited to the ProteomeXchange Consortium [42] *via* the PRIDE [43] partner repository with the dataset identifier PXD017198 and DOI: 10.6019/PXD017198.

Default PeptideShaker protein reports for each sample and quality controls are listed in Supplementary Tables S3–S5.

## Results and Discussion

The objectives of this study were to evaluate three different sample processing methods (the APFAR or DRP methods followed by in-solution digestion, or the SP3/HILIC method with magnetic bead-based digestion) as well as the effect of storage time (FFPE tissue block age) on protein extraction efficiency and reproducibility. Subsequent proteomic analysis by label-free LC-MS/MS evaluated the proteome coverage, proportion of missed cleavages, and enrichment/selection bias based on sample processing method used.

### Protein Extraction and Quantification

**FIGURE 3:**
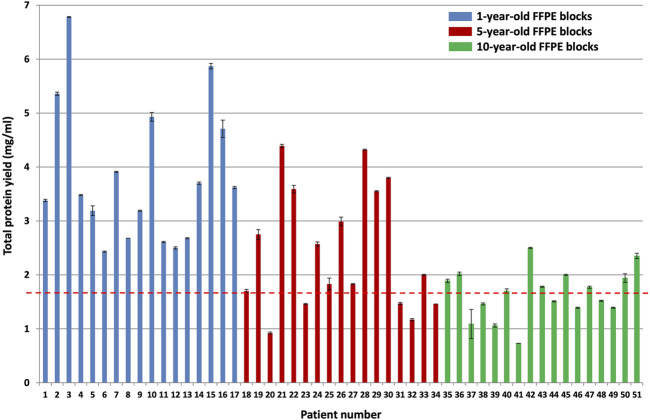
BCA total protein quantitation assay results for the different block ages. Protein was extracted from approximately 25 mm^3^ patient tumor tissue using 500 µl protein extraction buffer per sample (*n* = 17 patients per group, *p* < 0.0001). The blue bars indicate protein yield from 1-year-old FFPE blocks, the red bars indicate protein yield from 5-year-old FFPE blocks, and the green bars indicate protein yield from 10-year-old FFPE blocks. The red dotted line indicates the average protein yield obtained from the 10-year-old FFPE blocks, which is 1.65 mg/ml protein.

The BCA total protein quantitation assay results of all samples (after protein was extracted from approximately 25 mm^3^ patient tumor tissue using 500 µl of protein extraction buffer per sample) are shown in Figure 3.

A Kruskal–Wallis test was conducted to examine the differences in protein yield between block ages (Supplementary Table S2). Protein yield was significantly affected by block age (H (2) = 23.92, *p* < 0.0001), as seen in Figure 3. Based on Dunn’s post hoc testing results, there is evidence that the distribution of protein yields are significantly different for 1-year-old blocks vs 10-year-old blocks and for 1-year-old blocks vs 5-year-old blocks, but not for 5-year-old blocks vs 10-year-old blocks (results and conclusions are shown in Supplementary Table S2).

The 10-year-old FFPE tissues generated overall lower protein yields (an average of 1.65 ± 0.04 mg/ml) compared to the 5-year-old FFPE tissues, which generated an average of 2.46 ± 0.03 mg/ml protein, and the 1-year-old FFPE tissues, which generated an average of 3.82 ± 0.03 mg/ml protein. This corresponds to approximately 825 μg, 1,230 μg, and 1910 µg protein extracted from the 10, 5 and 1-year-old FFPE tissues, respectively, by using approximately 25 mm^3^ tissue per sample [14]. were able to extract 300–400 µg (0.14 mg/ml) protein from 1.18 mm^3^ FFPE colon adenoma tissue (of 60 µm thickness and 5 mm diameter), which is approximately 4 times higher. However, they noted a suppressive effect of formalin-fixation on protein yield estimates, using the BCA assay. This effect occurs because the amino acids that contribute to the reduction of copper are also susceptible to reactions with formaldehyde. Therefore, they empirically determined a correction factor for protein yield estimates of FFPE tissues (using the BCA assay) by comparing it to freshly frozen colon adenoma tissue replicates’ protein yields. They then used this correction factor to measure the amount of protein generated from their FFPE samples. Since we are not comparing fresh tissues to FFPE tissues, we did not determine the correction factor of our dataset and we report the protein yield estimates only. In addition [44], also extracted higher protein yields at 100 mg/ml protein from 0.1 mm^3^ FFPE colonic adenoma tissue and [13] extracted 2.76 mg/ml protein from approximately 1-year-old and 1.48 mg/ml protein from approximately 21-year-old (1.5 mm^3^) FFPE colon carcinoma tissue. On the other hand [45], extracted less protein than reported here, with 250 µg protein from approximately 18 mm^3^ FFPE colon carcinoma tissues that were stored for less than 5 years. Therefore, the amount of protein extracted here falls within the published ranges for FFPE colon tissue.

Although approximately 25 mm^3^ of manually microdissected tumor tissue per sample was used for protein extraction, and the volume of protein extraction buffer kept constant at 500 µl per sample, the total amount of extractable protein and protein yield still differed among the patient samples within the same block ages (Figure 3). Similar variations in protein yields were also observed by [13] and is also noted in FFPE protein extraction protocols, such as the [46] manual, which explains that protein yield obtained from FFPE protein lysates may vary between samples due to variance in pre-analytical factors such as tissue handling and inconsistencies/differences in the formalin-fixation and paraffin-embedment protocol, which affects how well proteins will be preserved. They recommend increasing the amount of starting material/tissue if the quality of protein preservation in the FFPE sample is questionable [46].

### The Effect of Block Age and Protein Purification Methods on Peptide and Protein Identification

**FIGURE 4:**
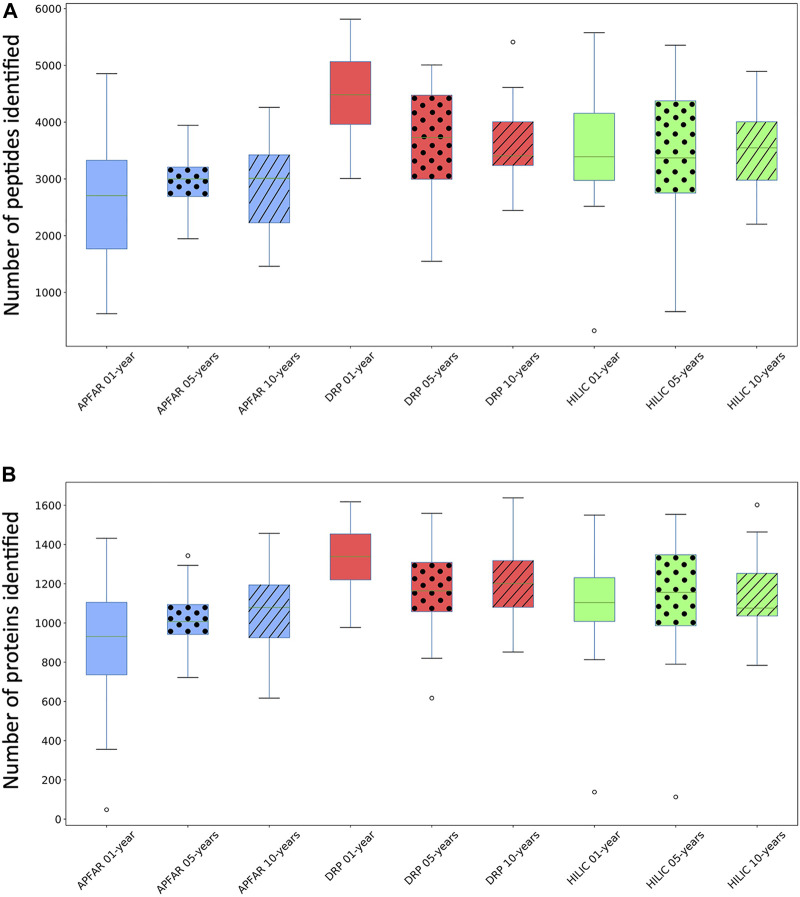
Comparison of the number of peptides and proteins identified for the different protein purification methods for each block age. **(A)** Box and whiskers plots of the number of peptides identified (for all 17 patient cases) per block age (*p* < 0.03 for 1 and 10-year-old blocks), and protein purification method (*p* = 0.0125 for DRP). **(B)** Box and whiskers plots of the number of proteins identified (for all 17 patient cases) per block age (*p* = 0.0002 for 1-year-old blocks) and protein purification method (*p* > 0.05 for all methods). Blue boxplots refer to APFAR samples; Red boxplots refer to DRP samples; Green boxplots refer to SP3/HILIC samples. For all boxplots, 5-year-old samples are represented by dots; 10-year-old samples are represented by diagonal lines.

The efficiency and reproducibility for each protein purification method, as well as the effect of storage time/block age, at both peptide and protein level, was assessed with regards to proteome coverage (number of peptides and proteins identified) (Figures 4A,B) and known protein biomarkers (proteins deregulated in colon cancer) from the literature, which were also identified in the data (Table 2).

**TABLE 2:** Known proteins deregulated in colon cancer.

					% Occurrence within 17 patient samples								
					APFAR			DRP			HILIC		
Main accession	Gene name	Protein name	MW (kDa)	Comments	1 year old	5 year old	10 year old	1 year old	5 year old	10 year old	1 year old	5 year old	10 year old
O95994	AGR2	Anterior gradient protein 2 homologue	19.97	Downregulated in CRC [47]	88	100	94	94	100	94	100	94	100
Q13951	CBFB	Core-binding factor subunit beta	21.49	Frequently overexpressed in CRC [48]	12	41	24	35	35	47	0	12	6
P08174	CD55; DAF	Complement decay-accelerating factor	41.37	Upregulated in CRC [49]	0	0	6	0	0	0	0	0	0
P10645	CHGA	Chromogranin-A	50.66	Downregulated in CRC [50]	29	29	18	18	18	18	24	18	18
A8K7I4	CLCA1	Calcium-activated chloride channel regulator 1	100.16	Regulator of calcium channels, frequently downregulated in CRC [51]	59	53	41	59	59	47	53	53	47
Q96KP4	CNDP2	Cytosolic non-specific dipeptidase	52.84	Overexpressed in CRC [52]	82	88	94	100	88	100	94	94	100
P07148	FABP1	FABP1 protein	14.20	Downregulated in CRC [47]	100	100	71	94	100	88	94	100	88
Q9Y6R7	FCGBP	IgGFc-binding protein	571.64	Downregulated in CRC [47]	76	94	82	76	94	76	82	88	82
P56470	LGALS4	Galectin-4	35.92	Downregulated in CRC [47]	100	100	100	100	100	100	94	100	100
P09429	HMGB1	High mobility group protein B1	24.88	Overexpression in CRC correlates with poor prognosis [53]	76	88	76	100	100	94	94	82	94
P01042	KNG1	Kininogen-1	71.91	Frequently overexpressed in CRC [54]	29	41	53	53	59	82	29	47	65
Q9UHB6	LIMA1	LIM domain and actin-binding protein 1	85.17	Downregulated in CRC [47]	0	0	6	0	0	24	6	6	0
P15941	MUC-1	Mucin-1	122.03	Frequently overexpressed in CRC, marker of poor prognosis [55]	0	6	12	6	6	12	0	6	6
Q02817	MUC-2	Mucin-2	539.96	Downregulation correlates with proliferation markers and with poor prognosis [55, 56]	59	59	76	71	65	71	65	71	76
P06748	NPM1	Nucleophosmin	32.55	Protein involved in carcinogenesis, overexpressed in CRC [57, 58]	100	100	100	100	100	100	100	100	100
Q6UX06	OLFM4	Olfactomedin-4	57.24	Protein overexpressed in CRC [54]	29	18	29	35	24	29	29	24	29
Q9Y617	PSAT1	Phosphoserine aminotransferase	40.40	Upregulated in CRC [59]	0	0	6	18	12	12	18	12	18
P53992	Sec24C	Protein transport protein Sec24C	118.25	Overexpressed in early CRC stages, while downregulated in advanced CRC stages [54]	0	0	0	0	6	6	6	0	0
P36952	SERPIN B5	Serpin B5	42.07	Upregulated in CRC [60]	29	6	29	35	6	29	29	6	29
P10599	TXN	Thioredoxin	11.73	Frequently overexpressed in CRC [61]	94	100	100	94	100	100	94	94	94

Average results for all samples (Figure 4) show that, overall, the DRP method performed the best with the highest overall peptide and protein identifications, followed by the SP3/HILIC method. The APFAR method generated the lowest numbers of peptide and protein identifications (Results are shown in Supplementary Table S6).

One-way ANOVA or Kruskal–Wallis tests were conducted (results and conclusions are listed in Supplementary Table S2) to determine if the number of identified peptides and proteins were significantly different between block ages, as well as for each protein purification method.

Statistical analyses comparing protein purification method performance per block age indicated the following: For the 1-year-old blocks, based on post hoc Bonferroni (Dunn) t tests, the DRP method differs significantly (F (2) = 12.78, *p* < 0.0001, *α* = 0.05) with regards to validated peptide identifications, however there was no significant difference between the numbers of validated peptides identified for the APFAR and SP3/HILIC methods. Based on Dunn’s post hoc testing results, there is also evidence that the distribution of validated protein identifications (for 1-year old blocks) are significantly different (*p* = 0.0002) for DRP vs APFAR processing, but not for DRP vs HILIC and APFAR vs HILIC protein purification methods. With regards to validated peptide and protein identifications, there is no significant difference (*p* > 0.05) between protein purification methods for 5-year old blocks. For the 10-year old blocks, based on post hoc Bonferroni (Dunn) t tests, the DRP and APFAR methods differ significantly (F (2) = 3.78, *p* = 0.0299, *α* = 0.05) with regards to validated peptide identifications, however there is no significant difference between the APFAR and SP3/HILIC and the DRP and SP3/HILIC methods.

Statistical analyses comparing the differences between block ages (effect of block age on the number of peptide/protein identifications) within each protein purification method indicated the following: Both the APFAR and SP3/HILIC methods performed most consistently across block ages, with no significant difference between 1, 5 and 10-year-old blocks [APFAR method: F (2,48) = 0.88, *p* = 0.42 for peptides identified and H (2) = 2.28, *p* = 0.32 for proteins identified; SP3/HILIC method: F (2,48) = 0.03, *p* = 0.97 for peptides identified and H (2) = 0.101, *p* = 0.95 for proteins identified]. Only the DRP method showed a significant difference between the block ages with regard to numbers of peptides identified [F (2) = 4.81, *p* = 0.0125, *α* = 0.05], with a significant difference between 1 and 5-year-old blocks, as well as 1 and 10-year-old blocks, but no significant difference between 5 and 10-year-old blocks. In addition, no significant difference was detected for the number of proteins identified [F (2,48) = 2.53, *p* = 0.09].

The protein purification methods that did not show any significant differences between block ages, are in accordance with the findings of other studies [10, 14]. [14] also assessed the effect of storage time/block age on FFPE colon adenoma tissue samples (stored for 1, 3, 5, or 10 years), using isoelectric focusing to fractionate peptides before LC-MS/MS analysis. They found no significant difference between the numbers of proteins identified for each block age and concluded that long-term storage of FFPE colon adenoma tissues did not compromise the samples. In general, the proteome coverage reported here (for all the block ages and protein purification methods) falls within the range of several other studies of proteomic analysis of FFPE tissue [3, 14, 62, 63], with higher identification numbers reported by other studies [10, 44, 64, 65]. Table 2 shows known proteins that are deregulated in colon cancer that were also identified in the data. The % occurrence of these proteins within each group of 17 patients per experimental condition was calculated and shows that there are no observable differences due to block age. However, the DRP method shows overall higher % occurrence of these protein biomarkers, compared to the other protein purification methods.

### The Effect of Block Age and Protein Purification Methods on Peptide-Level Reproducibility

The qualitative reproducibility for each sample and experimental condition was also measured in terms of peptide identification overlap (shown in Supplementary Image S3), calculated from the peptide sequences identified in each sample and experimental condition, irrespective of peptide abundance.

Supplementary Image S3A illustrates that the APFAR method showed the highest peptide overlap/common peptides (46.5%) between samples of different block ages. This was followed by the SP3/HILIC method, with 45.4% peptide overlap, and the lowest peptide overlap was seen for the DRP method at 43%. Overall, there was no substantial difference between uniquely identified peptides of the different block ages (ranging from 11.3% to 13.2%) for the APFAR and SP3/HILIC methods. However, the 1-year-old blocks processed with the DRP method had the highest percentage of uniquely identified peptides at 20.4%.

The shared peptides for each protein purification method within a specific block age are shown in Supplementary Image S3B. The 10-year-old blocks showed the highest peptide overlap/common peptides (37.5%) between the different protein purification methods. This was followed by the 5-year-old blocks, with 36.7% peptide overlap, and the lowest peptide overlap was seen for the 1-year-old blocks at 33.3%. This could be due, in part, to similar proteins extracted from the older blocks (since formaldehyde-induced cross-linking continues with time), compared to more diverse sets of proteins extracted from the more recently preserved 1-year-old blocks [66]. Due to the continuation and extent of formaldehyde-induced protein cross-linking with time, the extraction of full-length proteins from older FFPE blocks is also more difficult [66]. In addition [67], were able to identify small proteins, without antigen retrieval and enzymatic digestion steps, *via* mass spectrometry imaging. They hypothesize that not all proteins, especially small proteins (with short amino acid sequences and low lysine content), react with formaldehyde to the same extent. However, larger proteins (with longer amino acid sequences and greater lysine content) were more challenging to detect *via* mass spectrometry, and therefore have a greater probability of being more extensively crosslinked by formaldehyde. On average, for all block ages and protein purification methods, the identified proteins were in the range of 40–60 kDa (data not shown). This therefore indicates that mostly low and medium molecular weight proteins were extracted from the FFPE tissues at all block ages.

Supplementary Image S3C shows that, when all the identified peptides for each block age is combined within a protein purification method, there is 34.1% overlapping peptides shared between the different methods. The DRP method had the highest percentage of uniquely identified peptides at 19.5%, followed by the SP3/HILIC method, with 15.8% unique peptides, and the lowest uniquely identified peptides was seen for the APFAR method at 9.9%.

### Physicochemical Properties of Extracted and Processed Peptides

**FIGURE 5:**
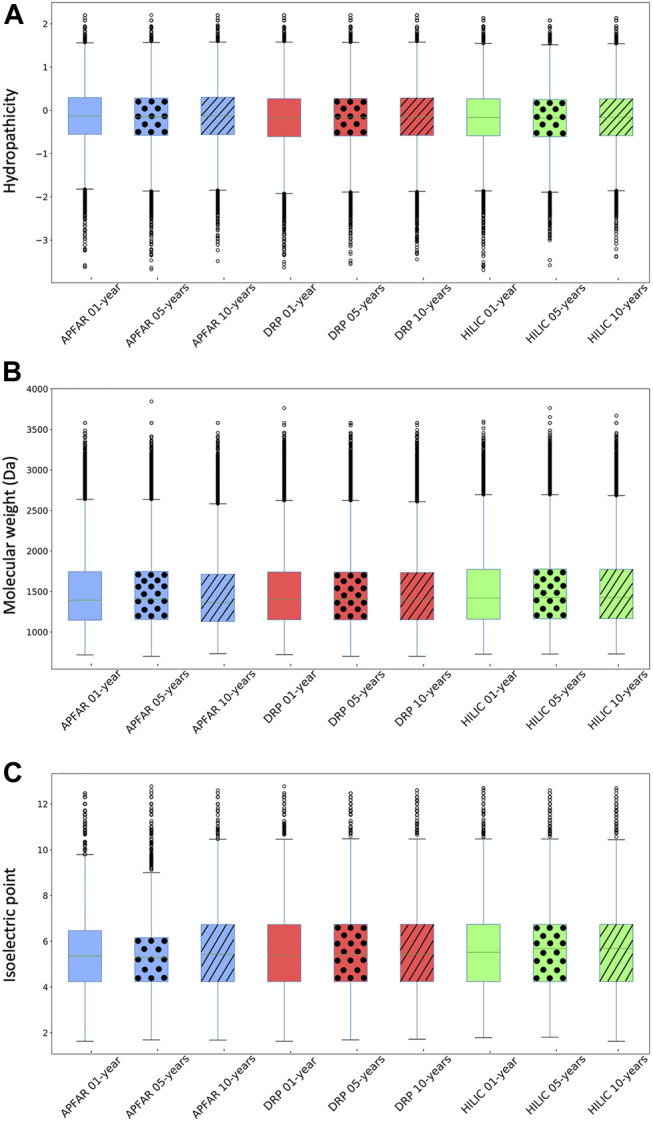
Physicochemical properties of identified peptides for all experimental conditions (*n* = 17 patients per group). **(A)** Hydropathicity based on GRAVY scoring matrix. **(B)** Molecular weight (MW). **(C)** Isoelectric point (pI). Blue boxplots refer to APFAR samples; Red boxplots refer to DRP samples; Green boxplots refer to HILIC samples. For all boxplots, 5-year-old samples are represented by dots; 10-year-old samples are represented by diagonal lines.

The effect of archival time/block age as well as protein purification method protein selection/enrichment bias was assessed with regards to peptide sequence physicochemical properties in Figure 5, which illustrates the peptide distribution according to hydropathicity, molecular weight and isoelectric point (pI). Kruskal–Wallis tests were conducted to determine if there were significant differences between experimental conditions (Supplementary Table S2).

Overall, a comparison of the majority (upper and lower quartiles) of all peptides of all experimental conditions shows that they share similar hydropathicity scales (Figure 5A). There is a significant difference (*p* < 0.0001) between the hydropathicity of peptides generated in all experimental conditions (Supplementary Table S2), however, the average relative hydropathicity of all the samples are negative (below zero), which indicates that the majority of peptides that were extracted and processed, by all three protein purification methods and across all block ages are hydrophilic [36, 68].

Figure 5B indicates that the molecular weight ranges of identified peptides are relatively constant across all samples and experimental conditions, with the majority >1000 Da and <2000 Da. There is a significant difference (*p* < 0.0001) between the molecular weights of peptides generated *via* the different protein purification methods for 1, 5 and 10-year old blocks. With regards to block age differences, there is no significant difference (*p* = 0.26) between the molecular weights of peptides generated *via* the DRP method, however there is a significant difference (*p* < 0.05) between the molecular weights of peptides generated using the APFAR and/or SP3/HILIC methods.

There is a significant difference (*p* < 0.0001) between the pI ranges of peptides generated in all experimental conditions, however, the pI range values are relatively similar across all samples and experimental conditions, with the majority above pI 4 and below pI 7 (Figure 5C).

These results are in accordance with previous studies that used the APFAR and SP3/HILIC methods [20–22, 69].

### The Effect of Block Age and Protein Purification Methods on Protein-Level Reproducibility

**FIGURE 6:**
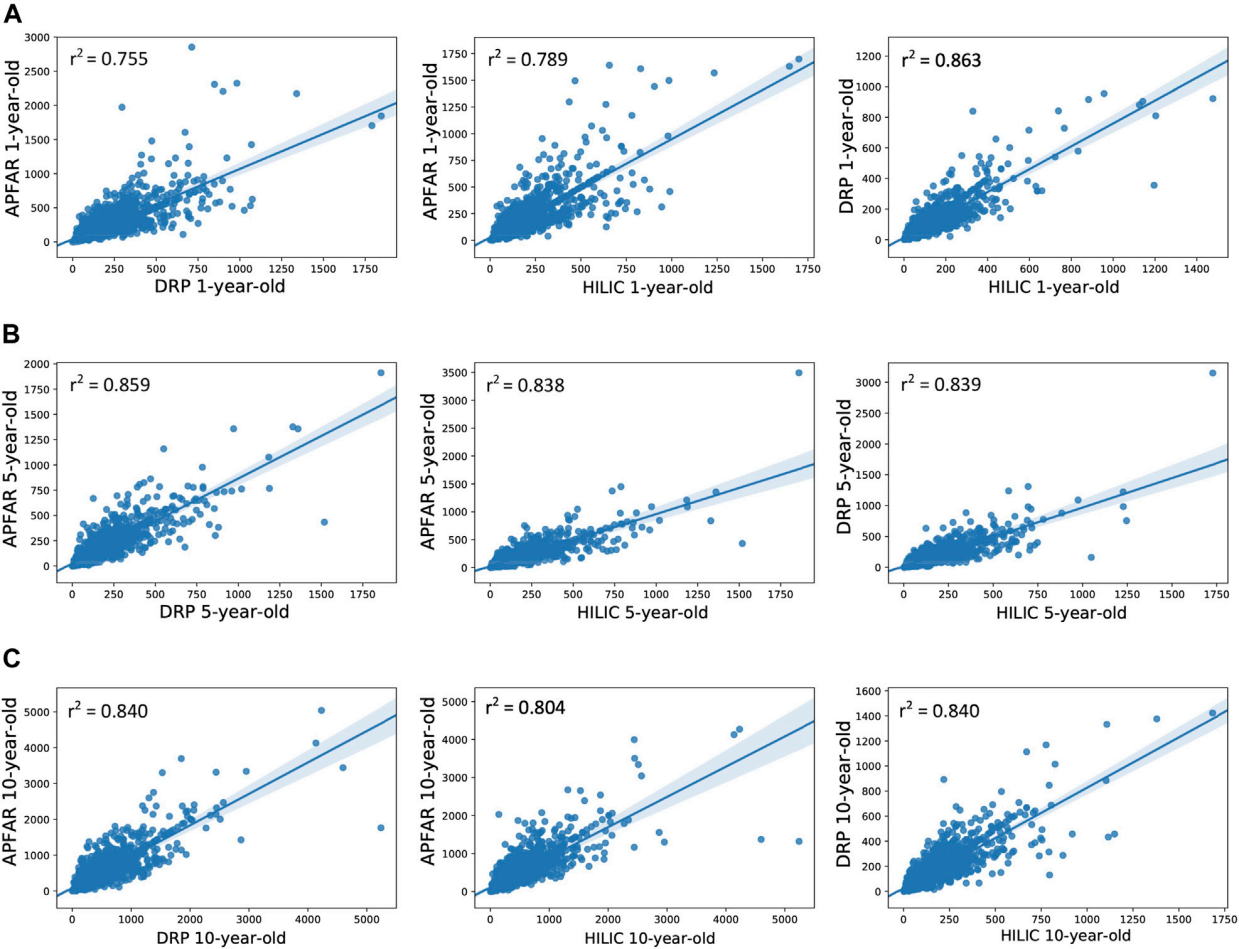
Correlation of protein abundance between all protein purification methods for each patient sample. **(A)** Correlation of protein abundance for all protein purification methods for 1-year-old blocks/samples (*n* = 17 patients per group). **(B)** Correlation of protein abundance for all protein purification methods for 5-year-old blocks/samples (*n* = 17 patients per group). **(C)** Correlation of protein abundance for all protein purification methods for 10-year-old blocks/samples (*n* = 17 patients per group). The Pearson correlation coefficients (*r* ^2^) are indicated on each plot and plot axes values are the normalized NSAF values for proteins present in both condition compared per plot.

**FIGURE 7:**
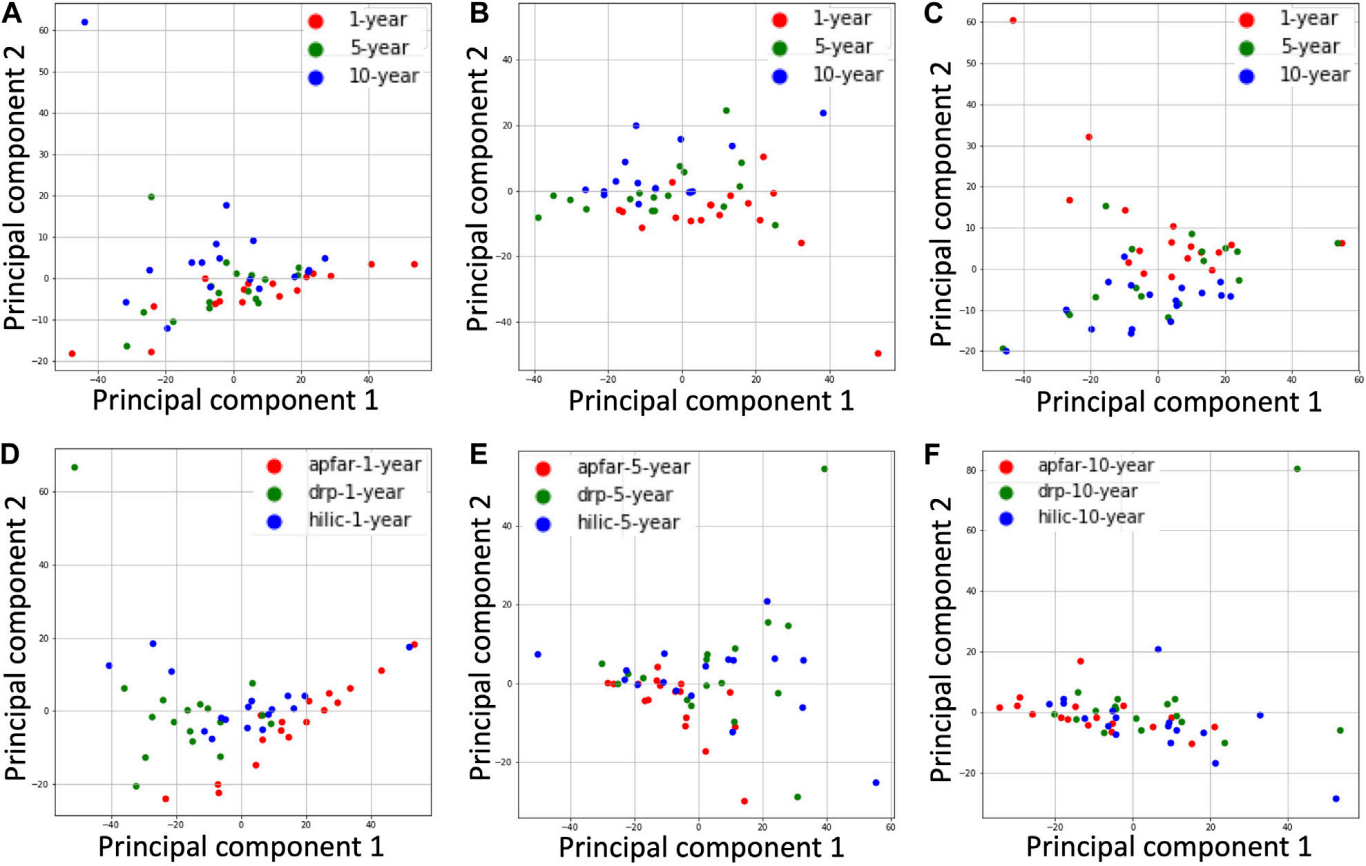
PCA plots for all block ages and protein purification methods. The NSAF values for proteins identified from each patient case were normalized and dimensionality reduced by principal component analysis of the datasets. **(A)** PCA plot of all block age (1-year-old = red; 5-year-old = green; 10-year-old = blue) samples processed via the APFAR method. **(B)** PCA plot of all block age (1-year-old = red; 5-year-old = green; 10-year-old = blue) samples processed via the DRP method. **(C)** PCA plot of all block age (1-year-old = red; 5-year-old = green; 10-year-old = blue) samples processed via the HILIC method. **(D)** PCA plot of 1-year-old samples for all protein purification methods (APFAR = red; DRP = green; HILIC = blue). **(E)** PCA plot of 5-year-old samples for all protein purification methods (APFAR = red; DRP = green; HILIC = blue). **(F)** PCA plot of 10-year-old samples for all protein purification methods (APFAR = red; DRP = green; HILIC = blue).

The quantitative reproducibility between experimental conditions were expressed as PCC dot plots (Figure 6), which were calculated based on the NSAF abundance values for identified proteins in each sample and experimental condition. PCA plots were also generated from this data to assess the variance between block ages and the protein purification methods (Figure 7).

Figure 6 shows the correlation of protein abundance for all protein purification methods for each block age. This illustrates that, for 1-year-old blocks, the DRP and SP3/HILIC methods yielded comparable relative protein abundances (PCC value of 0.863), whereas proteome composition correlation was lower for the AFFAR and DRP (PCC value of 0.755) as well as APFAR and SP3/HILIC (PCC value of 0.789) methods. Overall, the 5 and 10-year-old blocks show similar proteome composition correlation between the protein purification methods.

For 5-year-old blocks, the PCC values for the APFAR and SP3/HILIC, as well as DRP and SP3/HILIC methods are approximately equal, 0.838 and 0.839, respectively. The APFAR and DRP method has a higher PCC value of 0.859, indicating slightly higher correlation in proteome composition between these two protein purification methods.

For 10-year-old blocks, the PCC values for the APFAR and DRP as well as DRP and SP3/HILIC methods were the same. The APFAR and SP3/HILIC method has a lower PCC value of 0.804, indicating slightly lower correlation in proteome composition between these two protein purification methods. These results indicate that sample processing with the different methods introduces an observable bias with regard to proteome composition. This bias is also more pronounced for 1-year-old blocks, compared to older blocks.

PCA plots showing clusters of samples, based on their similarities, were generated for all block ages and protein purification methods (Figure 7). The samples that have similar expression profiles are clustered together. Figures 7A–C show the clustering of different block ages (1, 5, and 10 years) for each protein purification method, with the DRP method having the lowest variance (10.73%) between block ages, followed by the SP3/HILIC method (13.68%), and the APFAR method, which has the highest variance at 14.57%.

For the protein purification methods (Figures 7D–F), the 10-year-old blocks/samples shows the lowest variance between the different methods (11.4%), followed by the 5-year-old blocks/samples. This could be due, in part, to similar proteins extracted from the older blocks because the formaldehyde-induced protein cross-linking process is continual and becomes more extensive with time [66] (also noted and discussed in *The Effect of Block Age and Protein Purification Methods on Peptide-Level Reproducibility*). The 1-year-old blocks/samples (Figure 7D) shows the highest variance (15.86%) between the different methods.

### GO Analysis of Identified Proteins

**FIGURE 8:**
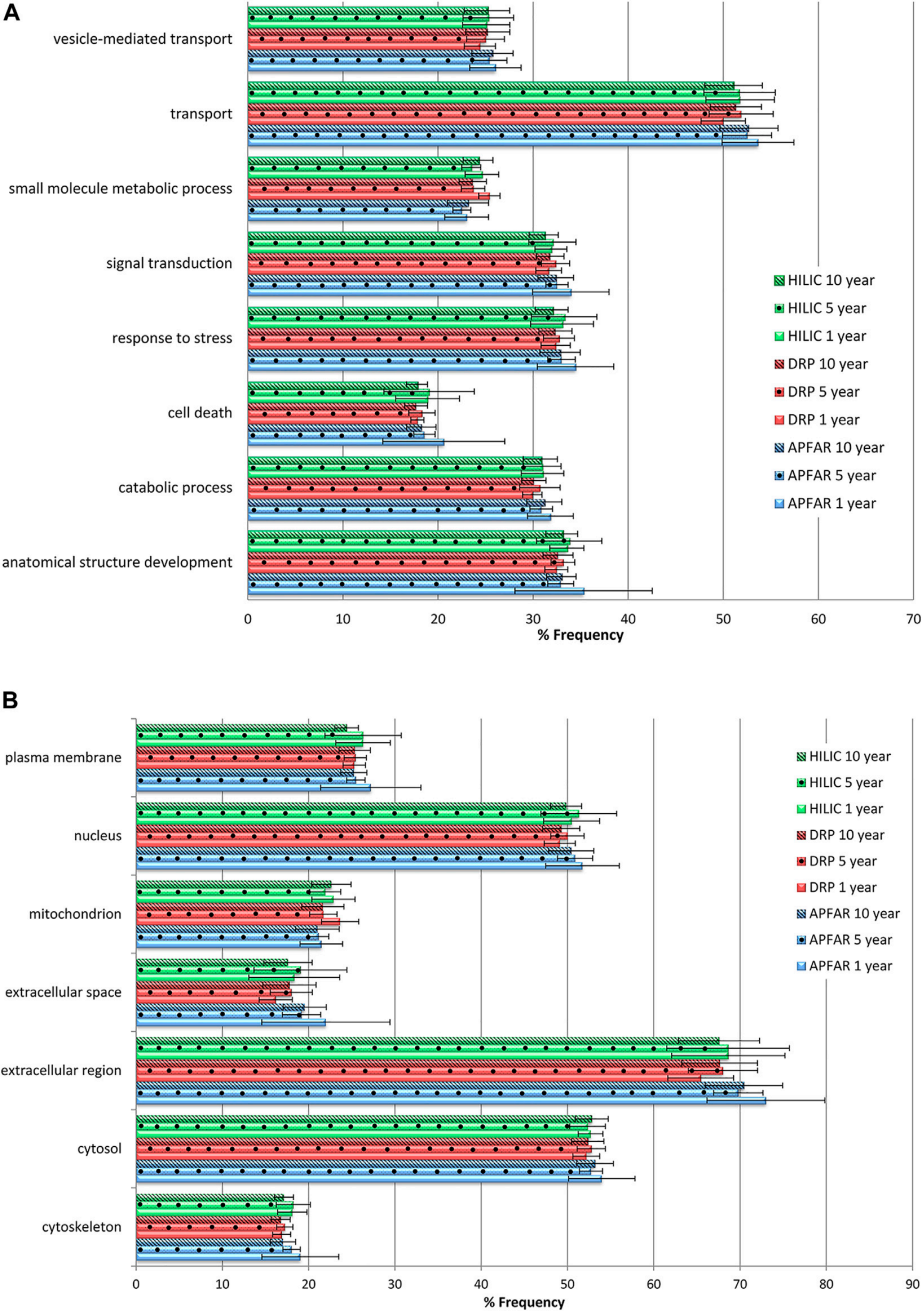
Gene Ontology annotation profiles for proteins identified from all block ages and protein purification methods. **(A)** GO profiles according to biological processes. **(B)** GO profiles according to cellular components. The average proportions for all 17 patients per condition are shown with error bars indicating the standard deviation. Blue bars refer to APFAR samples; Red bars refer to DRP samples; Green bars refer to HILIC samples. For all samples, 5-year-old samples are represented by dots; 10-year-old samples are represented by diagonal lines.

The effect of storage time/block age as well as the protein purification methods’ protein selection biases were assessed with regards to the main biological processes and cellular components present within the identified proteins, using Gene Ontology (GO) annotation. The distribution of the percentages of proteins belonging to each GO term was plotted for GO terms that occurred at >15% frequency for all samples and experimental conditions (Figure 8).

Overall, similar GO profiles were obtained for all samples, therefore only the GO terms that showed some observable difference between experimental conditions were plotted. Figure 8A shows the percentage frequency at which the identified proteins (of all experimental conditions) occurs for each of the plotted GO terms for biological processes, and Figure 8B shows cellular components.

One-way ANOVA or Kruskal–Wallis tests were conducted (results are listed in Supplementary Table S2) to determine if there were any significant differences with regard to the % frequency of occurrence of GO terms between block ages, as well as for each protein purification method.

All GO terms (for all block ages and protein purification methods) occurred at >15% frequency for all samples and are clearly represented by Figure 8. Therefore all block ages and protein purification methods used in this study demonstrate more or less equivalent usability for proteomic analysis. Statistically significant differences were mainly observed for 1-year-old blocks processed *via* the APFAR and/or DRP methods. Overall, the HILIC method showed least bias across all GO terms plotted.

Statistical analyses for protein purification methods showed that some GO terms for 1-year-old blocks processed *via* the APFAR and DRP methods were significantly enriched (*p* < 0.05) for % frequency of occurrence when using the APFAR method. These GO terms were: catabolic process, cytoskeleton, extracellular region, extracellular space, mitochondrion and transport. The APFAR method differed significantly (depleted) (*p* < 0.05) from both the DRP and HILIC methods for 1 and 5-year-old blocks for % frequency of occurrence of the term “small molecule metabolic process”.

Statistical analyses for the different block ages (processed *via* the DRP method) showed that the 1-year-old blocks are significantly enriched (*p* < 0.05) for GO terms “mitochondrion” and “small molecule metabolic process” when compared to the 5 and 10-year-old blocks. In addition, for samples processed *via* the HILIC method, only the 10-year-old blocks were significantly depleted (*p* < 0.05) for % frequency of occurrence of the term “plasma membrane” when compared to the 1 and 5-year-old blocks. For samples processed *via* the APFAR method, the 1-year-old blocks were significantly enriched (*p* < 0.05) whereas the 10-year-old blocks were significantly depleted (*p* < 0.05) with regard to the GO term “cytoskeleton”.

### Assessment of the Digestion Efficiency of the Protein Purification Methods for all Block Ages

To assess the reproducibility and digestion efficiency of the different protein purification methods, the percentages of missed cleavages across all samples were analyzed (shown in Supplementary Image S4). To successfully analyze FFPE tissues requires overcoming the issue of the formaldehyde cross-linking between molecules [2, 4, 6, 11]. The most important aspect to take into consideration for accurate protein extraction from FFPE tissues is the cleavage of these methylene bridges to allow for proper trypsin digestion. The methylene bridges prevent trypsin from reaching its cleavage sites. If the methylene bridges are not adequately cleaved, it will result in improperly digested, cross-linked peptides that will not produce correct MS results. Therefore, the effect of storage time/block age on trypsin digestion efficiency was also determined by comparing the percentage of missed cleavages across all block ages.

Supplementary Image S4 shows that overall, all protein purification methods and all block ages generated low numbers of missed cleavages. The APFAR method (Supplementary Image S4A) generated the lowest percentages of missed cleavages with ≥85% of all peptides for 1-year-old samples having no missed cleavages, and ≥90% of all peptides for 5 and 10-year-old samples having no missed cleavages. This was followed by the DRP method (Supplementary Image S4B), with ≥85% of all peptides (except for sample number DRP-9) for 1-year-old samples having no missed cleavages, and ≥85% of all peptides for 5 and 10-year-old samples having no missed cleavages (except for sample number DRP-34 of the 5-year-old cohort). The SP3/HILIC method (Supplementary Image S4C) had overall lower digestion efficiency with ≥80% of all peptides for 1 and 5-year-old samples having no missed cleavages, and ≥80% of all peptides for 10-year-old samples having no missed cleavages (except for samples HILIC-37 and HILIC-41).

The protein purification methods’ digestion efficiency therefore does not appear to be only affected by the age of the sample, since older and newer blocks gave varying results depending on the processing method used [14]. found that after deparaffinization and rehydration, cross-linked proteins are efficiently digested with trypsin, without the need for additional specialized reagents, even under mild conditions typically used for fresh tissues. This is also observed here, since all block ages and protein purification methods used demonstrate sufficient trypsin activity/cleavage efficiency, with all samples showing low levels of missed cleavages. Generally, the percentage of missed cleavages of the present study was in the range of several other recent reports [69–71], with lower percentage of missed cleavages reported in [20].

### Effects of Block Age and Protein Purification Methods on Sample Proteome Integrity

The oxidation of methionine is a major protein modification, which converts methionine to methionine sulfoxide, and targets the affected protein for degradation, both *in vivo* and *in vitro* [72]. Methionine oxidation is linked to processes relating to aging and pathology (*in vivo*) as well as *in vitro* conditions caused by protein purification, storage, light exposure, and exposure to free radicals generated in the presence of metals during LC-MS/MS analysis [72]. To determine the impact of long-term storage, the percentage of peptides containing methionine oxidation (out of the total number of peptides identified) was calculated for all block ages and protein purification methods (Supplementary Image S5).

Kruskal–Wallis tests were conducted to determine if the percentage of peptides containing methionine oxidation were significantly different between block ages for each protein purification method (Supplementary Table S2). No significant differences were found between 1, 5 and 10-year old blocks/samples processed *via* the APFAR [H (2) = 1.23, *p* = 0.54], DRP [H (2) = 0.86, *p* = 0.65], or SP3/HILIC [H (2) = 3.38, *p* = 0.18] methods. Supplementary Image S5 shows that for the 10-year-old blocks/samples the percentage of peptides with methionine oxidation are 8.77 ± 3.41%, 7.77 ± 2.41%, and 5.47 ± 2.13%, for APFAR, DRP and SP3/HILIC respectively. Similar percentages of peptides with methionine oxidation (7.65 ± 2.05% and 7.38 ± 2.15%) are observed for 1 and 5-year-old blocks/samples processed *via* the APFAR method. The same is seen for 1 and 5-year-old blocks/samples processed *via* the DRP method (7.24 ± 2.11% and 6.83 ± 1.69%). The SP3/HILIC method has lower percentages of peptides with methionine oxidation for all block ages, with 4.34 ± 1.36%, 4.43 ± 1.16% and 5.47 ± 2.13%, for 1, 5, and 10-year-old blocks/samples respectively. Therefore, the choice of sample preparation/protein purification method may contribute to methionine oxidation artifacts [72]. [73] found that methionine oxidation increases during enzymatic digestion, with the presence of residual metals in the digestion buffer, sample contact with metal surfaces, as well as chromatography separation.

The SP3/HILIC method’s results are in agreement with results reported by [3] for newly preserved (<1-year-old) FFPE samples (processed using acetone precipitation and sodium hydroxide resolubilization for protein purification), which had methionine oxidation ratios of 3.9–4.5% for all identified peptides. In contrast [14], reported higher methionine oxidation levels and found that archived colon adenoma tissues displayed an increase in methionine oxidation with block age - from 16.8% after one year of storage, 18.2% for 5-year-old samples up to 25.2% after 10 years of storage.

## Conclusion

Archived FFPE tissue repositories are precious sources of clinical material, often stored for decades, for clinical proteomic studies. Since these preserved blocks may be conveniently stored at ambient temperatures, it makes them easily accessible and cost effective. However, standardized protocols for the proteomic analysis of FFPE tissues have not been determined yet. In addition, the effect of block age and storage at resource-limited institutions, on protein quality remains unclear. We have demonstrated, using recently developed protein purification techniques (and FFPE human colorectal cancer resection tissues) that, overall, block age mainly affects protein yields during the protein extraction phase. Therefore, greater amounts of starting material are required for older blocks prior to LC-MS/MS analysis. Analyzed samples’ peptide and protein identifications mainly differed according to the protein purification method used and not block age, which mainly impacted on tissue proteome composition.

This study is also of particular relevance, since it assessed the performance of three different protein purification techniques on tissues derived from samples stored over a long period of time (1–10 years). The comparative analyses of these methods, across different block ages, have not been carried out to our knowledge and therefore this study provides both experimental data for this assessment as well as statistical support. The different methods show differences in the number of peptides and proteins identified and sample proteome composition, differences in reproducibility in terms of peptide identification overlap, PCA variance, as well as protocol digestion efficiency. Overall, the DRP and SP3/HILIC methods performed the best, with the SP3/HILIC method requiring less protein (and therefore less starting material) than the other methods, therefore making it the most sensitive and efficient protein purification method.

These results are encouraging since they indicate that long-term storage of FFPE tissues does not significantly interfere with retrospective proteomic analysis. In addition, variations in pre-analytical factors (spanning a decade), such as tissue harvesting, handling, the fixation protocol used as well as storage conditions (at resource-limited institutions in developing countries), does not affect protein extraction and shotgun proteomic analysis to a significant extent.

## Data Availability

The mass spectrometry proteomics data [41] generated and analyzed for this study can be found in the PRIDE [43] repository (http://www.ebi.ac.uk/pride/archive/) with the dataset identifier PXD017198 and DOI: 10.6019/PXD017198.

## Footnotes

1
www.ensembl.org

2
www.ebi.ac.uk/GOA

## Glossary

ACN: Acetonitrile
AmBic: Ammonium bicarbonate
APFAR: Acetone precipitation and formic acid resolubilization
BCA: Bicinchoninic acid
CRC: colorectal carcinoma
DRP: detergent removal plates
ESI: electrospray ionization
FA: Formic acid
FDR: False discovery rate
FFPE: Formalin-fixed, paraffin-embedded
GO: Gene ontology
H&E: Hematoxylin and Eosin
HIAR: Heat-induced antigen retrieval
HILIC: Hydrophilic interaction liquid chromatography
LC: Liquid chromatography
LC-MS/MS: Liquid chromatography coupled to tandem mass spectrometry
LFQ: Label-free quantitation
MMTS: Methylmethanethiosulfonate
MS: Mass spectrometry
NSAF: Normalized spectrum abundance factor
PCA: Principal component analysis
PCC: Pearson’s correlation coefficient
PSM: Peptide Spectrum Match
PTM(s): Post-translational modification(s)
SDS: Sodium dodecyl sulfate
SP3: Single-Pot Solid-Phase-enhanced Sample Preparation
TCEP: Tris(2-carboxyethyl)phosphine
TEAB: Triethylammonium bicarbonate
TFA: Trifluoroacetic acid
