# Supplementary material for: Evaluation of Protein Purification Techniques and Effects of Storage Duration on LC-MS/MS Analysis of Archived FFPE Human CRC Tissues
Source: Pathol Oncol Res. 2021 May 3;27:622855. doi: 10.3389/pore.2021.622855 (PMC8262168; doi:10.3389/pore.2021.622855)
Supplement: Supplementary file 1 [file DataSheet2.PDF]

Supplementary Material

Supplementary Image 2 – Graphical PeptideShaker output of an example of post-translational modification localizations (oxidation of methionine) for a peptide

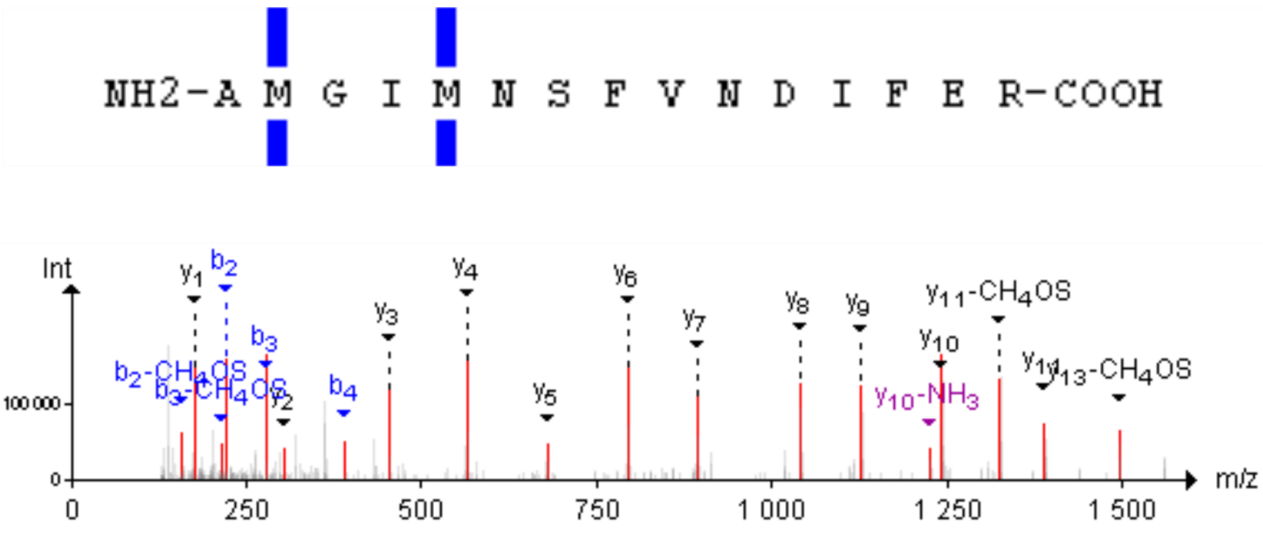

Supplementary Figure 2. Graphical PeptideShaker output of an example of post-translational modification localizations (oxidation of methionine) for a peptide.
