# Supplementary material for: Evaluation of Protein Purification Techniques and Effects of Storage Duration on LC-MS/MS Analysis of Archived FFPE Human CRC Tissues
Source: Pathol Oncol Res. 2021 May 3;27:622855. doi: 10.3389/pore.2021.622855 (PMC8262168; doi:10.3389/pore.2021.622855)
Supplement: Supplementary file 2 [file Table1.DOCX]

Supplementary Material

Supplementary Data Sheet 1 – Algorithms specific settings and certificate of analysis (PeptideShaker output)

Project Details

1: PeptideShaker Version: 1.16.40
2: Date:
3: Experiment:
4: Sample:
5: Replicate Number: 1
6: Identification Algorithms: X!Tandem, MS Amanda and MS-GF+

Database Search Parameters

1: Precursor Tolerance Unit: ppm
2: Precursor Ion m/z Tolerance: 10.0
3: Fragment Ion Tolerance Unit: Da
4: Fragment Ion m/z Tolerance: 0.02
5: Cleavage: Enzyme
6: Enzyme: Trypsin
7: Missed Cleavages: 2
8: Specificity: Specific
9: Database: uniprot-human-reviewed-trypsin-may-2018_concatenated_target_decoy.fasta
10: Forward Ion: b
11: Rewind Ion: y
12: Fixed Modifications: Methylthio of C
13: Variable Modifications: Oxidation of M, Deamidation of N, Deamidation of Q
14: Refinement Variable Modifications: Acetylation of protein N-term, Pyrolidone from E, Pyrolidone from Q, Pyrolidone from carbamidomethylated C
15: Refinement Fixed Modifications: Methylthio of C

Input Filters

1: Minimal Peptide Length: 8
2: Maximal Peptide Length: 30
3: Precursor m/z Tolerance: 10.0
4: Precursor m/z Tolerance Unit: Yes
5: Unrecognized Modifications Discarded: Yes

PTM Scoring Settings

1: Probabilistic Score: PhosphoRS
2: Accounting for Neutral Losses: No
3: Threshold: 95.0

Spectrum Counting Parameters

1: Method: NSAF
2: Validated Matches Only: No

Annotation Settings

1: Intensity Limit: 0.75
2: Automatic Annotation: Yes
3: Selected Ions: y, b
4: Neutral Losses: H2O, NH3, CH4OS
5: Neutral Losses Sequence Dependence: Yes
6: Fragment Ion m/z Tolerance: 0.02
