# Supplementary material for: Evaluation of Protein Purification Techniques and Effects of Storage Duration on LC-MS/MS Analysis of Archived FFPE Human CRC Tissues
Source: Pathol Oncol Res. 2021 May 3;27:622855. doi: 10.3389/pore.2021.622855 (PMC8262168; doi:10.3389/pore.2021.622855)
Supplement: Supplementary file 3 [file Table8.DOCX]

Supplementary Material

*Supplementary Image 3: Comparison of the qualitative reproducibility of the experimental conditions in terms of peptide identification overlap for block ages and protein purification methods.*

*Supplementary Image 4: The numbers of missed cleavages for all block ages and protein purification methods.*

*Supplementary Image 5: Percentages of peptides containing oxidized methionine for all block ages and protein purification methods.*


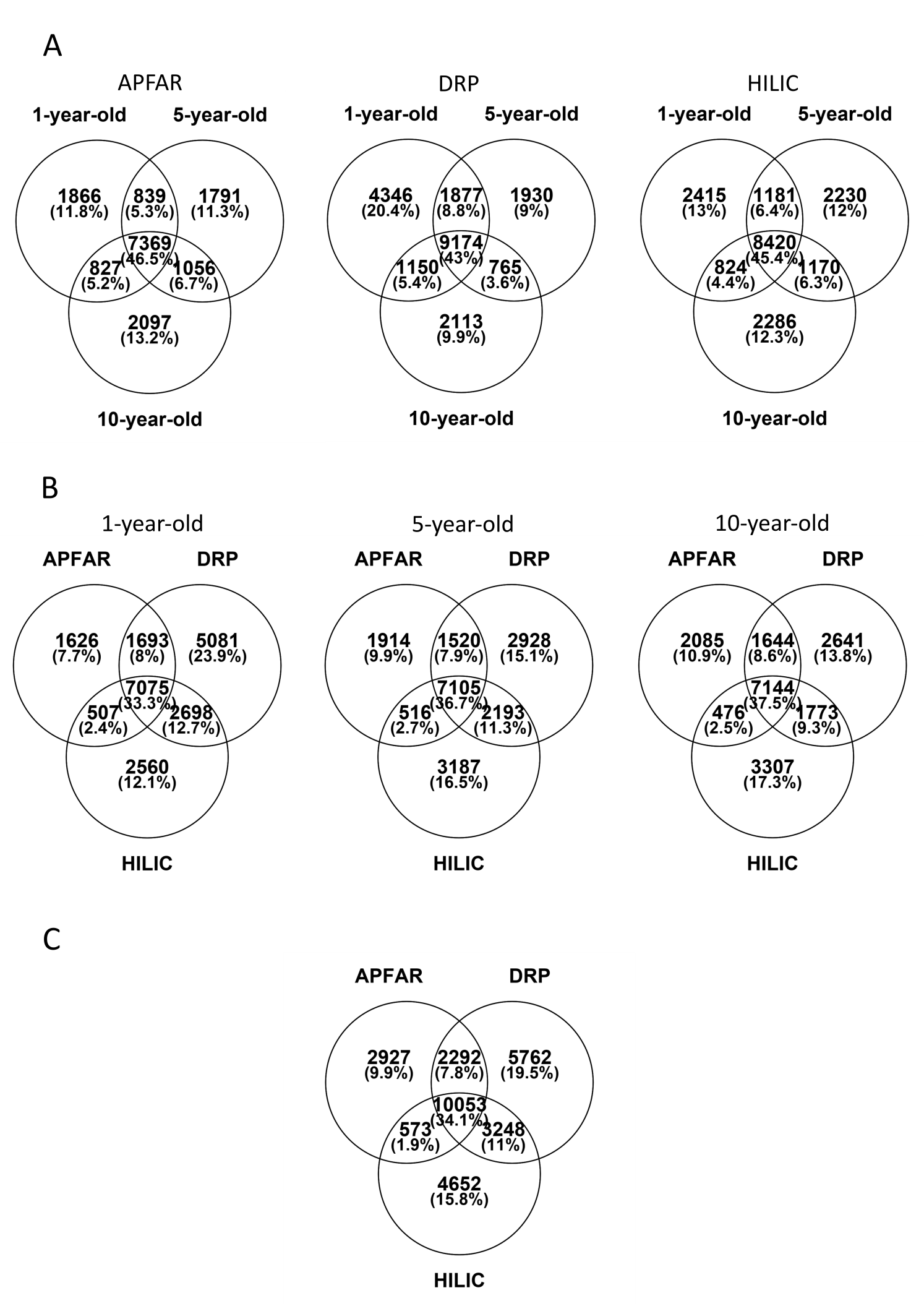


**Supplementary Image 3. Comparison of the qualitative reproducibility of the experimental conditions in terms of peptide identification overlap for block ages and protein purification methods.** **(A)** Venn diagrams depicting the distribution of identified peptides for 1, 5 and 10-year-old blocks within each protein purification method (*n* = 17 patients per group) **(B)** Venn diagrams depicting the distribution of identified peptides for each protein purification method within each block age (*n* = 17 patients per group) **(C)** Venn diagrams showing the overlap of combined identified peptides (of all block ages) for each protein purification method (*n* = 51).


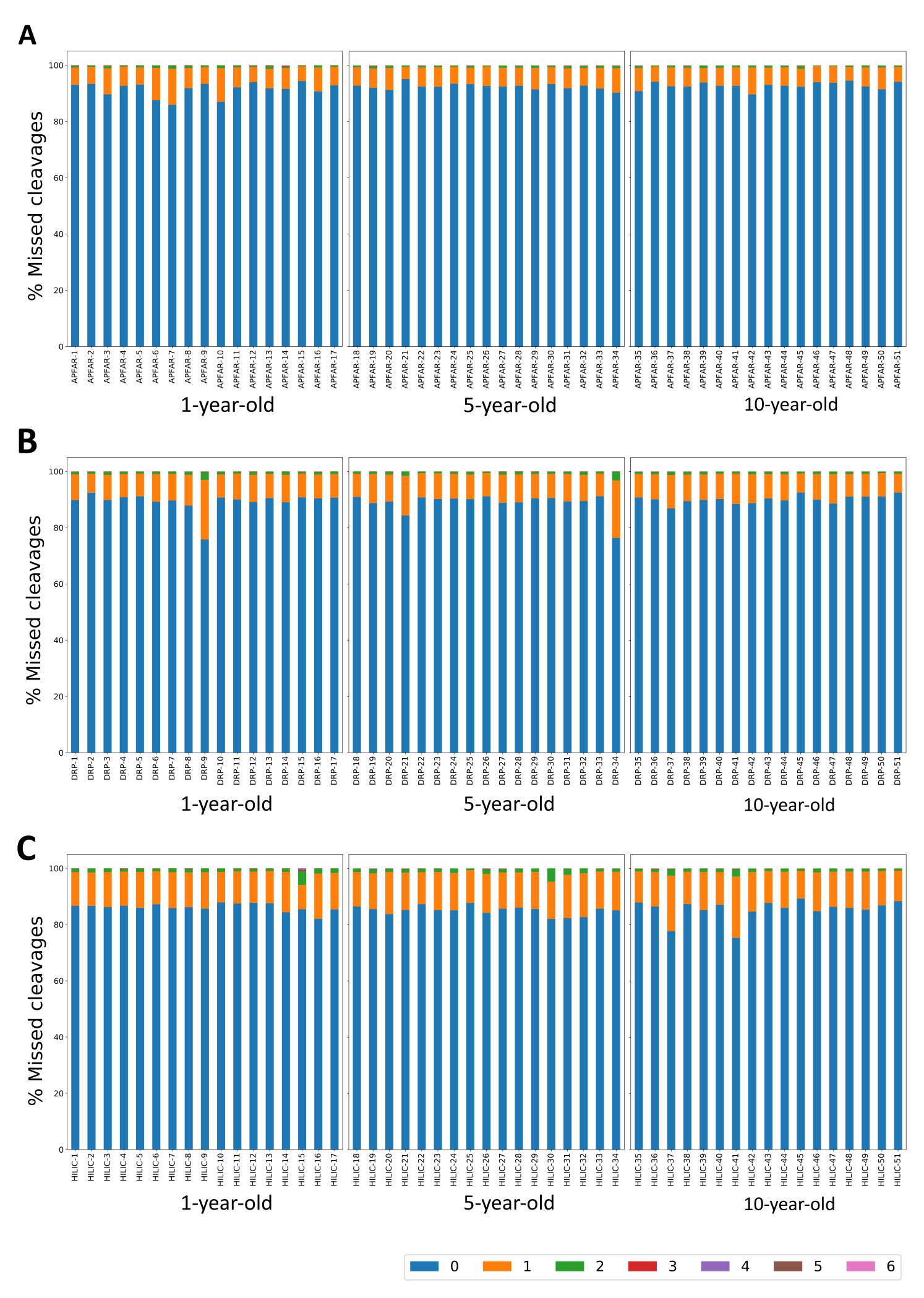


**Supplementary Image 4. The numbers of missed cleavages for all block ages and protein purification methods.** For each protein purification method and block age (*n* = 17 patients per group), the percentages of missed cleavages are plotted. **(A)** APFAR 1, 5 and 10-year-old blocks **(B)** DRP 1, 5 and 10-year-old blocks **(C)** SP3/HILIC 1, 5 and 10-year-old blocks. The figure key in the bottom right corner shows the graph colors for corresponding number of missed cleavages, with 0 missed cleavages = blue, 1 missed cleavage = orange, 2 missed cleavages = green, etc.


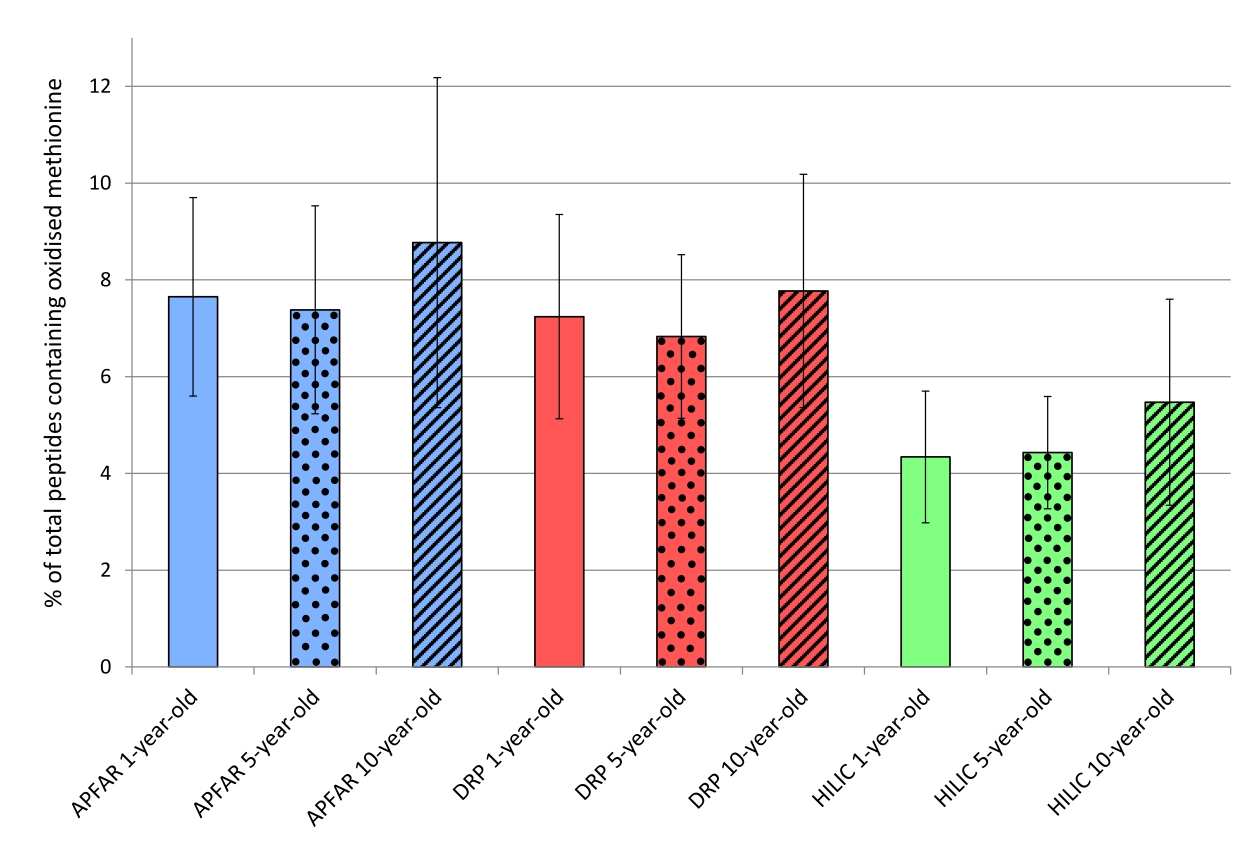


**Supplementary Image 5. Percentages of peptides containing oxidized methionine for all block ages and protein purification methods.** The percentages of peptides containing oxidized methionine, relative to the total number of identified peptides, were calculated for each patient sample analyzed (*n* = 17, p > 0.05 for all) per block age and protein purification method, and the averages plotted here. Error bars refer to the standard deviation. Blue bars refer to APFAR samples; Red bars refer to DRP samples; Green bars refer to HILIC samples. For all bars, 5-year-old samples are represented by dots; 10-year-old samples are represented by diagonal lines.
