# Supplementary material for: Evaluation of Protein Purification Techniques and Effects of Storage Duration on LC-MS/MS Analysis of Archived FFPE Human CRC Tissues
Source: Pathol Oncol Res. 2021 May 3;27:622855. doi: 10.3389/pore.2021.622855 (PMC8262168; doi:10.3389/pore.2021.622855)
Supplement: Supplementary file 4 [file DataSheet3.PDF]

Supplementary Material

Supplementary Image 3: Comparison of the qualitative reproducibility of the experimental conditions in terms of peptide identification overlap for block ages and protein purification methods.

Supplementary Image 4: The numbers of missed cleavages for all block ages and protein purification methods.

Supplementary Image 5: Percentages of peptides containing oxidized methionine for all block ages and protein purification methods.

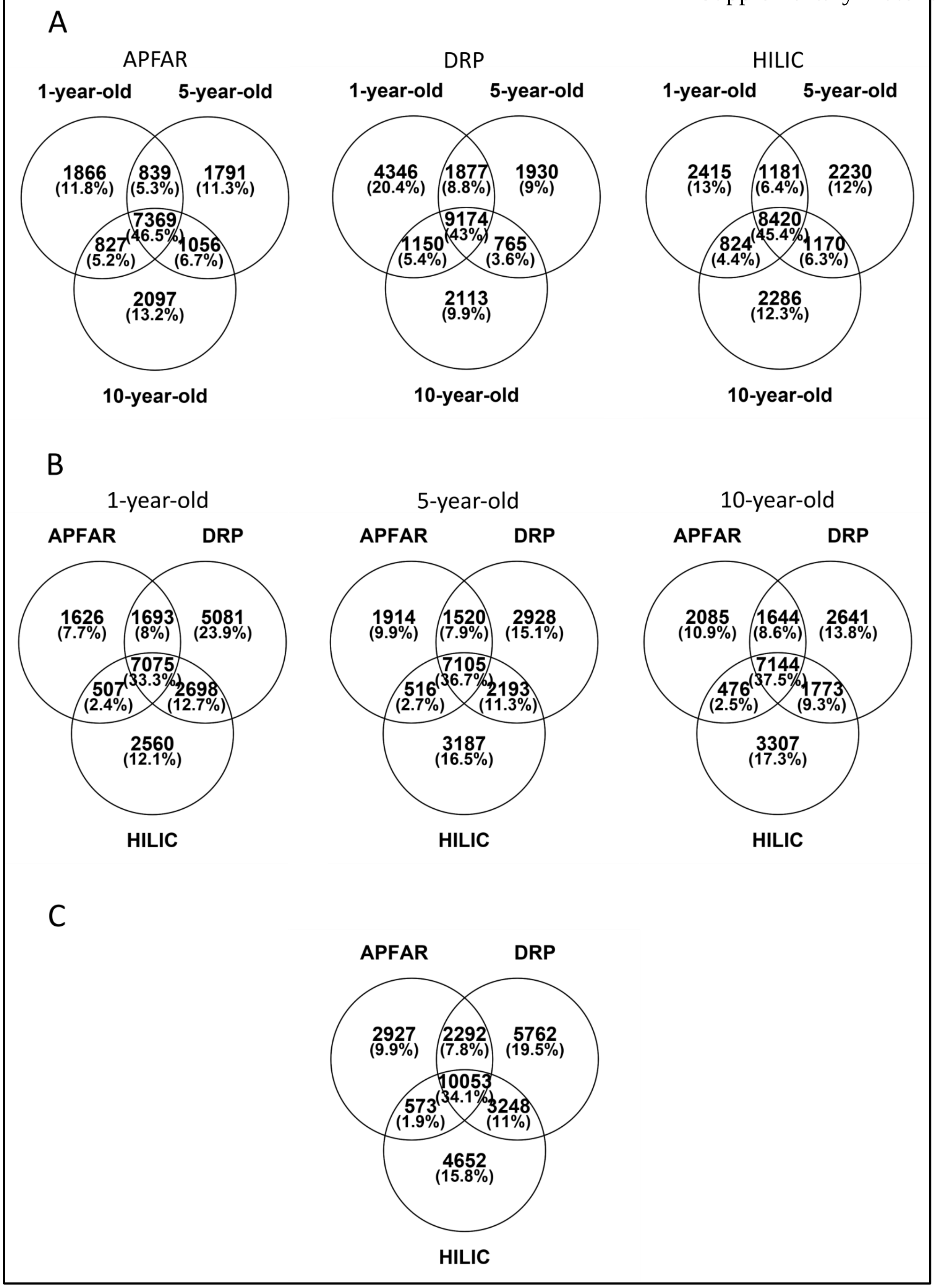

Supplementary Image 3. Comparison of the qualitative reproducibility of the experimental conditions in terms of peptide identification overlap for block ages and protein purification methods. (A) Venn diagrams depicting the distribution of identified peptides for 1, 5 and 10-year-old blocks within each protein purification method (*n* = 17 patients per group) **(B)** Venn diagrams depicting the distribution of identified peptides for each protein purification method within each block age (*n* = 17 patients per group) **(C)** Venn diagrams showing the overlap of combined identified peptides (of all block ages) for each protein purification method (*n* = 51).

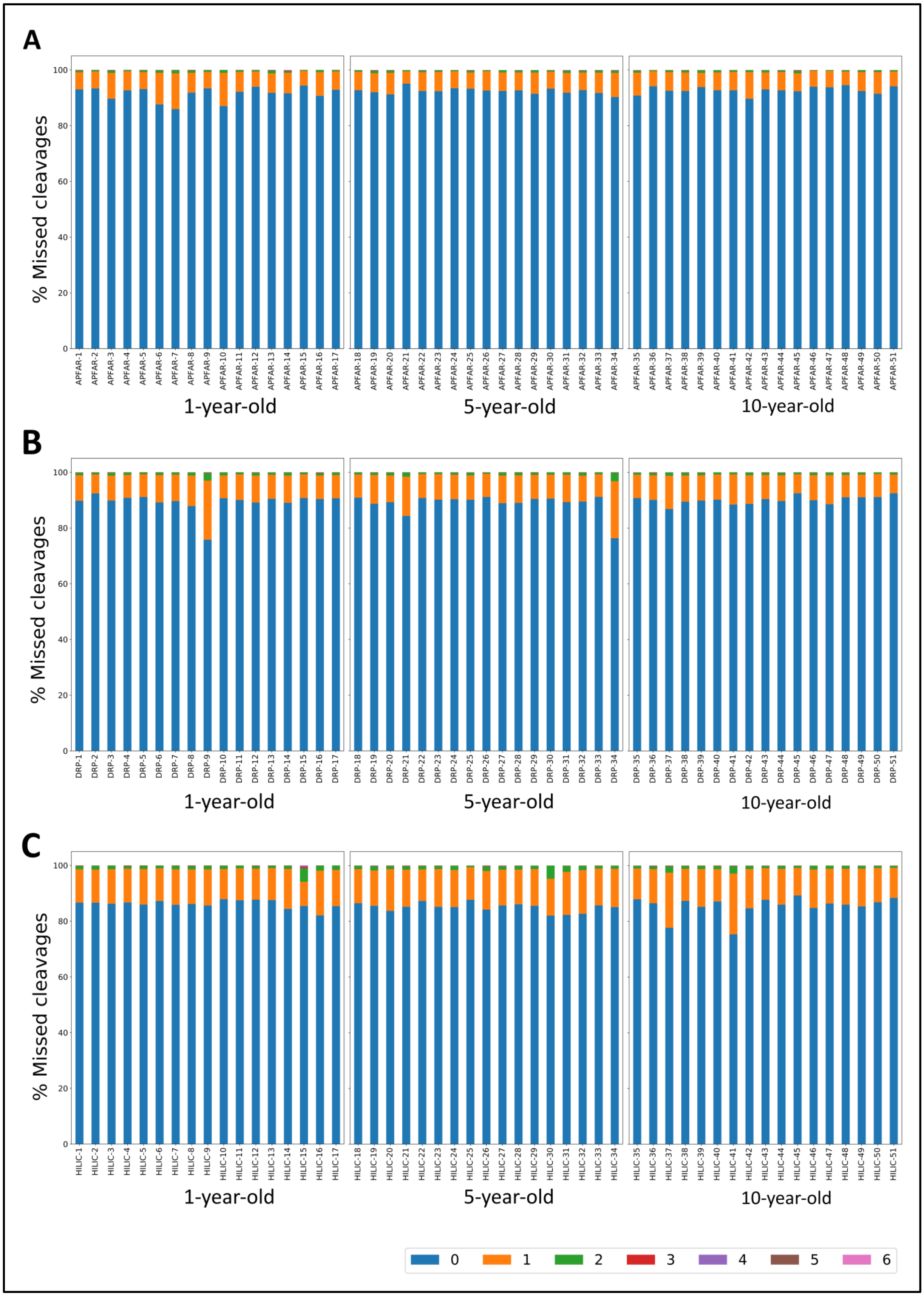

Supplementary Image 4. The numbers of missed cleavages for all block ages and protein purification methods. For each protein purification method and block age (n = 17 patients per group), the percentages of missed cleavages are plotted. **(A)** APFAR 1, 5 and 10-year-old blocks **(B)** DRP 1, 5 and 10-year-old blocks **(C)** SP3/HILIC 1, 5 and 10-year-old blocks. The figure key in the bottom right corner shows the graph colors for corresponding number of missed cleavages, with 0 missed cleavages = blue, 1 missed cleavage = orange, 2 missed cleavages = green, etc.

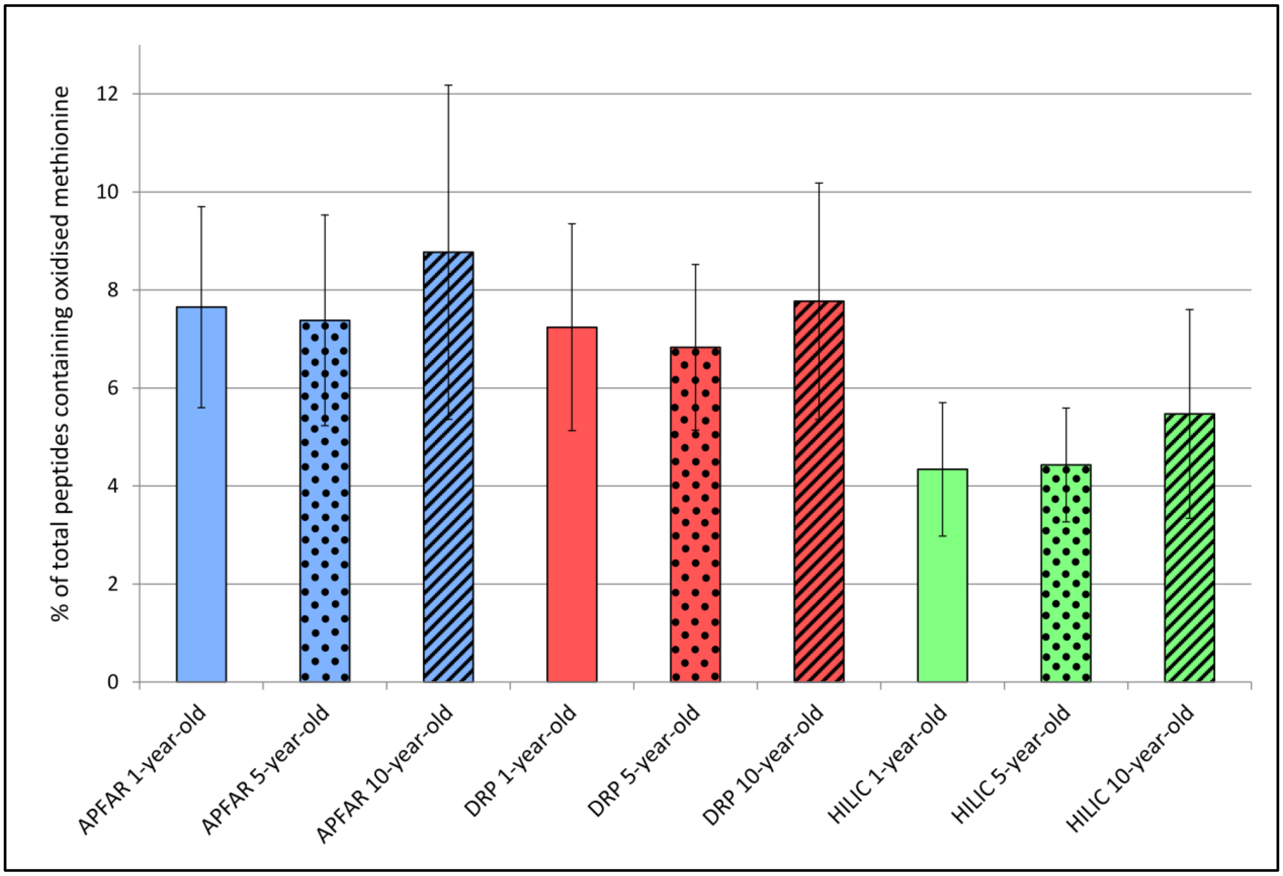

Supplementary Image 5. Percentages of peptides containing oxidized methionine for all block ages and protein purification methods. The percentages of peptides containing oxidized methionine, relative to the total number of identified peptides, were calculated for each patient sample analyzed (n = 17, $p > 0.05$ for all) per block age and protein purification method, and the averages plotted here. Error bars refer to the standard deviation. Blue bars refer to APFAR samples; Red bars refer to DRP samples; Green bars refer to HILIC samples. For all bars, 5-year-old samples are represented by dots; 10-year-old samples are represented by diagonal lines.
