# Supplementary material for: Evaluation of Protein Purification Techniques and Effects of Storage Duration on LC-MS/MS Analysis of Archived FFPE Human CRC Tissues
Source: Pathol Oncol Res. 2021 May 3;27:622855. doi: 10.3389/pore.2021.622855 (PMC8262168; doi:10.3389/pore.2021.622855)
Supplement: Supplementary file 6 [file Table2.DOCX]

Supplementary Material

Supplementary Table 1 – Details of data acquisition parameters

Supplementary Table 1. Details of data acquisition parameters.

| **Software function:** | **Parameter:** | **Value:** |
| --- | --- | --- |
| **Full scan** | Resolution | 70,000 (@ *m/z* 200) |
|  | AGC target value | 3e6 |
|  | Scan range | 350 – 2,000 *m/z* |
|  | Maximal injection time | 100 ms |
| **Data-dependent MS/MS** | Inclusion | Off |
|  | Resolution | 17,500 (@ *m/z* 200) |
|  | AGC target value | 1e5 |
|  | Maximal injection time | 50 ms |
|  | Loop count | 10 |
|  | Isolation window width | 3 Da |
|  | NCE | 27% |
| **Data-dependent settings** | Underfill ratio | 1% |
|  | Charge exclusion | Unassigned, 1, 7, 8, >8 |
|  | Peptide match | Preferred |
|  | Exclusion isotopes | On |
|  | Dynamic exclusion | 60 s |
