# Supplementary material for: Evaluation of Protein Purification Techniques and Effects of Storage Duration on LC-MS/MS Analysis of Archived FFPE Human CRC Tissues
Source: Pathol Oncol Res. 2021 May 3;27:622855. doi: 10.3389/pore.2021.622855 (PMC8262168; doi:10.3389/pore.2021.622855)
Supplement: Supplementary file 7 [file DataSheet1.PDF]

Supplementary Material

Supplementary Image 1 – Graphical PeptideShaker output of an example of an annotated MS/MS spectrum for a peptide

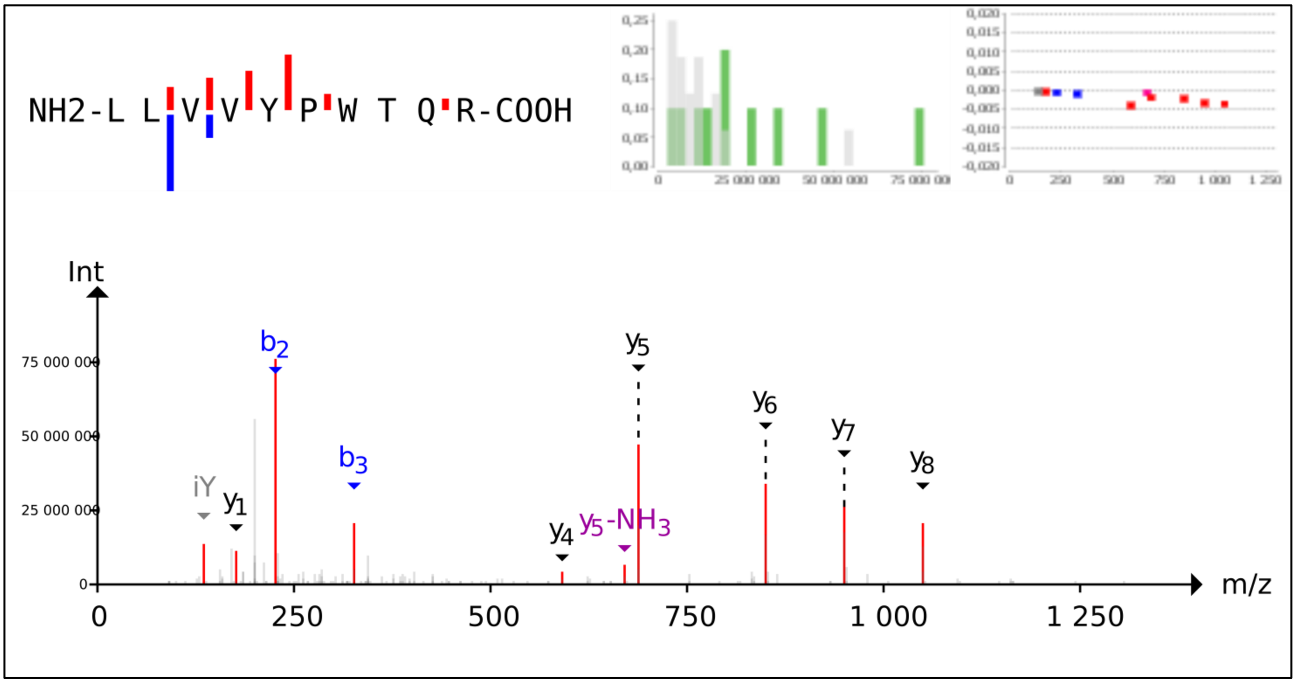

Supplementary Figure 1. Graphical PeptideShaker output of an example of an annotated MS/MS spectrum for a peptide.
