# Supplementary material for: Evaluation of Protein Purification Techniques and Effects of Storage Duration on LC-MS/MS Analysis of Archived FFPE Human CRC Tissues
Source: Pathol Oncol Res. 2021 May 3;27:622855. doi: 10.3389/pore.2021.622855 (PMC8262168; doi:10.3389/pore.2021.622855)
Supplement: Supplementary file 9 [file Table3.DOCX]

Supplementary Material

Supplementary Table 2 – Statistical tests information

Supplementary Table 2. Statistical tests information.

| **Data analyzed:** | **Statistical tests:** | **Conclusions:** |
| --- | --- | --- |
| Figure 3:  BCA total protein quantitation assay | 1. Shapiro–Wilk test:  W = 0.921, p = 0.002  2. Kruskal–Wallis test:  H(2) = 23.92, p < 0.0001  3. Dunn's post hoc test, α = 0.05 | 1. p < 0.05 therefore the distribution is significantly different from a normal distribution, i.e. it is non-normal.  2. Protein yield was significantly affected by block age, H(2) = 23.92, p < 0.05.  3. Protein yields from 1-year-old blocks vs 10-year-old blocks: there is evidence (at α = 0.05) to reject the null hypothesis (that the groups are equal/there is no significant difference). Therefore, there is significant location differences between groups. Protein yields from 1-year-old blocks vs 5-year-old blocks: there is evidence (at α = 0.05) to reject the null hypothesis. Therefore, there is significant location differences between groups. Protein yields from 5-year-old blocks vs 10-year-old blocks: there is evidence (at α = 0.05) to accept the null hypothesis. Therefore, there is no significant location differences between groups. |
| Figure 4 A: Comparison of the number of peptides identified for different protein purification methods for 1-year-old blocks | 1. Shapiro–Wilk test:  W = 0.979, p = 0.48  2. One-way ANOVA:  F(2,48) = 12.78, p < 0.0001  3. Bonferroni (Dunn) t Tests for validated peptides identified: F(2) = 12.78, p <0.0001 (α = 0.05) | 1. p > 0.05 therefore the distribution is not significantly different from a normal distribution, i.e. it is normal.  2. With regards to peptide identifications, there is a significant difference (p < 0.05) between protein purification/sample preparation methods for 1-year old blocks.  3. Compared to the other protein purification methods, the DRP method differs significantly with regards to peptide identifications for 1-year-old-blocks. There is no significant difference between the APFAR and SP3/HILIC methods. |
| Figure 4 A: Comparison of the number of peptides identified for different protein purification methods for 5-year-old blocks | 1. Shapiro–Wilk test:  W = 0.987, p = 0.83  2. One-way ANOVA:  F(2,48) = 1.51, p = 0.23 | 1. p > 0.05 therefore the distribution is not significantly different from a normal distribution, i.e. it is normal.  2. With regards to peptide identifications, there is not a significant difference (p > 0.05) between protein purification/sample preparation methods for 5-year old blocks. |
| Figure 4 A: Comparison of the number of peptides identified for different protein purification methods for 10-year-old blocks | 1. Shapiro–Wilk test:  W = 0.994, p = 0.99  2. One-way ANOVA:  F(2,48) = 3.78, p = 0.03  3. Bonferroni (Dunn) t Tests for validated peptides identified: F(2) = 3.78, p = 0.0299 (α = 0.05) | 1. p > 0.05 therefore the distribution is not significantly different from a normal distribution, i.e. it is normal.  2. With regards to peptide identifications, there is a significant difference (p < 0.05) between protein purification/sample preparation methods for 10-year old blocks.  3. The DRP and APFAR methods differ significantly with regards to peptide identifications for 10-year-old-blocks. There is no significant difference between the APFAR and SP3/HILIC and the DRP and SP3/HILIC methods. |
| Figure 4 A: Comparison of the number of peptides identified for different block ages for the APFAR method | 1. Shapiro–Wilk test:  W = 0.982, p = 0.65  2. One-way ANOVA:  F(2,48) = 0.88, p = 0.42 | 1. p > 0.05 therefore the distribution is not significantly different from a normal distribution, i.e. it is normal.  2. With regards to peptide identifications, there is not a significant difference (p > 0.05) between block ages when processed with the APFAR method. |
| Figure 4 A: Comparison of the number of peptides identified for different block ages for the DRP method | 1. Shapiro–Wilk test:  W = 0.988, p = 0.90  2. One-way ANOVA:  F(2,48) = 4.81, p = 0.01  3. Bonferroni (Dunn) t Tests for validated peptides identified: F(2) = 4.81, p = 0.0125 (α = 0.05) | 1. p > 0.05 therefore the distribution is not significantly different from a normal distribution, i.e. it is normal.  2. With regards to peptide identifications, there is a significant difference (p < 0.05) between block ages when processed with the DRP method.  3. With regards to peptide identifications, there is a significant difference (p < 0.05) between 1-year-old blocks and 5-year-old blocks, as well as 1-year-old blocks and 10-year-old blocks processed via the DRP method. There is no significant difference between 5 and 10-year-old blocks. |
| Figure 4 A: Comparison of the number of peptides identified for different block ages for the HILIC method | 1. Shapiro–Wilk test:  W = 0.970, p = 0.22  2. One-way ANOVA:  F(2,48) = 0.03, p = 0.97 | 1. p > 0.05 therefore the distribution is not significantly different from a normal distribution, i.e. it is normal.  2. With regards to peptide identifications, there is not a significant difference (p > 0.05) between block ages when processed with the HILIC method. |
| Figure 4 B: Comparison of the number of proteins identified for different protein purification methods for 1-year-old blocks | 1. Shapiro–Wilk test:  W = 0.924, p = 0.003  2. Kruskal–Wallis test:  H(2) = 16.70, p = 0.0002  3. Dunn's post hoc test, α = 0.05 | 1. p < 0.05 therefore the distribution is significantly different from a normal distribution, i.e. it is non-normal.  2. With regards to protein identifications, there is a significant difference (p < 0.05) between protein purification/sample preparation methods for 1-year old blocks.  3. For 1-year old blocks, protein identifications from DRP vs APFAR processing: there is evidence (at α = 0.05) to reject the null hypothesis (that the groups are equal/there is no significant difference). Therefore, there is significant location differences between these groups. For DRP vs HILIC and APFAR vs HILIC: there is evidence (at α = 0.05) to accept the null hypothesis. Therefore, there is no significant location differences between these groups. |
| Figure 4 B: Comparison of the number of proteins identified for different protein purification methods for 5-year-old blocks | 1. Shapiro–Wilk test:  W = 0.950, p = 0.03  2. Kruskal–Wallis test:  H(2) = 3.58, p = 0.17 | 1. p < 0.05 therefore the distribution is significantly different from a normal distribution, i.e. it is non-normal.  2. With regards to protein identifications, there is not a significant difference (p > 0.05) between protein purification/sample preparation methods for 5-year old blocks. |
| Figure 4 B: Comparison of the number of proteins identified for different protein purification methods for 10-year-old blocks | 1. Shapiro–Wilk test:  W = 0.991, p = 0.97  2. One-way ANOVA:  F(2,48) = 2.44, p = 0.098 | 1. p > 0.05 therefore the distribution is not significantly different from a normal distribution, i.e. it is normal.  2. With regards to protein identifications, there is not a significant difference (p > 0.05) between protein purification/sample preparation methods for 10-year old blocks. |
| Figure 4 B: Comparison of the number of proteins identified for different block ages for the APFAR method | 1. Shapiro–Wilk test:  W = 0.951, p = 0.03  2. Kruskal–Wallis test:  H(2) = 2.28, p = 0.32 | 1. p < 0.05 therefore the distribution is significantly different from a normal distribution, i.e. it is non-normal.  2. With regards to protein identifications, there is not a significant difference (p > 0.05) between block ages when processed with the APFAR method. |
| Figure 4 B: Comparison of the number of proteins identified for different block ages for the DRP method | 1. Shapiro–Wilk test:  W = 0.983, p = 0.69  2. One-way ANOVA:  F(2,48) = 2.53, p = 0.09 | 1. p > 0.05 therefore the distribution is not significantly different from a normal distribution, i.e. it is normal.  2. With regards to protein identifications, there is no significant difference (p > 0.05) between block ages when processed with the DRP method. |
| Figure 4 B: Comparison of the number of proteins identified for different block ages for the HILIC method | 1. Shapiro–Wilk test:  W = 0.894, p = 0.0003  2. Kruskal–Wallis test:  H(2) = 0.101, p = 0.95 | 1. p < 0.05 therefore the distribution is significantly different from a normal distribution, i.e. it is non-normal.  2. With regards to protein identifications, there is not a significant difference (p > 0.05) between block ages when processed with the HILIC method. |
| Figure 6 A: Comparison of the hydropathicity of identified peptides for different protein purification methods for 1-year-old blocks | 1. Kolmogorov-Smirnov test: D = 0.013, p < 0.01  2. Kruskal–Wallis test:  H(2) = 124.67, p < 0.0001 | 1. p < 0.05 therefore the distribution is significantly different from a normal distribution, i.e. it is non-normal.  2. There is a significant difference (p < 0.05) between the hydropathicity of peptides generated via the different protein purification/sample preparation methods for 1-year old blocks. |
| Figure 6 A: Comparison of the hydropathicity of identified peptides for different protein purification methods for 5-year-old blocks | 1. Kolmogorov-Smirnov test: D = 0.012, p < 0.01  2. Kruskal–Wallis test:  H(2) = 78.92, p < 0.0001 | 1. p < 0.05 therefore the distribution is significantly different from a normal distribution, i.e. it is non-normal.  2. There is a significant difference (p < 0.05) between the hydropathicity of peptides generated via the different protein purification/sample preparation methods for 5-year old blocks. |
| Figure 6 A: Comparison of the hydropathicity of identified peptides for different protein purification methods for 10-year-old blocks | 1. Kolmogorov-Smirnov test: D = 0.012, p < 0.01  2. Kruskal–Wallis test:  H(2) = 67.39, p < 0.0001 | 1. p < 0.05 therefore the distribution is significantly different from a normal distribution, i.e. it is non-normal.  2. There is a significant difference (p < 0.05) between the hydropathicity of peptides generated via the different protein purification/sample preparation methods for 10-year old blocks. |
| Figure 6 A: Comparison of the hydropathicity of identified peptides for different block ages for the APFAR method | 1. Kolmogorov-Smirnov test: D = 0.013, p < 0.01  2. Kruskal–Wallis test:  H(2) = 30.61, p < 0.0001 | 1. p < 0.05 therefore the distribution is significantly different from a normal distribution, i.e. it is non-normal.  2. There is a significant difference (p < 0.05) between the hydropathicity of peptides generated from 1, 5 and 10-year old blocks when using the APFAR method. |
| Figure 6 A: Comparison of the hydropathicity of identified peptides for different block ages for the DRP method | 1. Kolmogorov-Smirnov test: D = 0.013, p < 0.01  2. Kruskal–Wallis test:  H(2) = 55.79, p < 0.0001 | 1. p < 0.05 therefore the distribution is significantly different from a normal distribution, i.e. it is non-normal.  2. There is a significant difference (p < 0.05) between the hydropathicity of peptides generated from 1, 5 and 10-year old blocks when using the DRP method. |
| Figure 6 A: Comparison of the hydropathicity of identified peptides for different block ages for the HILIC method | 1. Kolmogorov-Smirnov test: D = 0.013, p < 0.01  2. Kruskal–Wallis test:  H(2) = 39.49, p < 0.0001 | 1. p < 0.05 therefore the distribution is significantly different from a normal distribution, i.e. it is non-normal.  2. There is a significant difference (p < 0.05) between the hydropathicity of peptides generated from 1, 5 and 10-year old blocks when using the HILIC method. |
| Figure 6 B: Comparison of the molecular weights of identified peptides for different protein purification methods for 1-year-old blocks | 1. Kolmogorov-Smirnov test: D = 0.085, p < 0.01  2. Kruskal–Wallis test:  H(2) = 94.28, p < 0.0001 | 1. p < 0.05 therefore the distribution is significantly different from a normal distribution, i.e. it is non-normal.  2. There is a significant difference (p < 0.05) between the molecular weights of peptides generated via the different protein purification/sample preparation methods for 1-year old blocks. |
| Figure 6 B: Comparison of the molecular weights of identified peptides for different protein purification methods for 5-year-old blocks | 1. Kolmogorov-Smirnov test: D = 0.085, p < 0.01  2. Kruskal–Wallis test:  H(2) = 138.67, p < 0.0001 | 1. p < 0.05 therefore the distribution is significantly different from a normal distribution, i.e. it is non-normal.  2. There is a significant difference (p < 0.05) between the molecular weights of peptides generated via the different protein purification/sample preparation methods for 5-year old blocks. |
| Figure 6 B: Comparison of the molecular weights of identified peptides for different protein purification methods for 10-year-old blocks | 1. Kolmogorov-Smirnov test: D = 0.086, p < 0.01  2. Kruskal–Wallis test:  H(2) = 488.53, p < 0.0001 | 1. p < 0.05 therefore the distribution is significantly different from a normal distribution, i.e. it is non-normal.  2. There is a significant difference (p < 0.05) between the molecular weights of peptides generated via the different protein purification/sample preparation methods for 10-year old blocks. |
| Figure 6 B: Comparison of the molecular weights of identified peptides for different block ages for the APFAR method | 1. Kolmogorov-Smirnov test: D = 0.089, p < 0.01 2. Kruskal–Wallis test:  H(2) = 208.75, p < 0.0001 | 1. p < 0.05 therefore the distribution is significantly different from a normal distribution, i.e. it is non-normal.  2. There is a significant difference (p < 0.05) between the molecular weights of peptides generated from 1, 5 and 10-year old blocks when using the APFAR method. |
| Figure 6 B: Comparison of the molecular weights of identified peptides for different block ages for the DRP method | 1. Kolmogorov-Smirnov test: D = 0.084, p < 0.01  2. Kruskal–Wallis test:  H(2) = 2.71, p = 0.26 | 1. p < 0.05 therefore the distribution is significantly different from a normal distribution, i.e. it is non-normal.  2. There is no significant difference (p < 0.05) between the molecular weights of peptides generated from 1, 5 and 10-year old blocks when using the DRP method. |
| Figure 6 B: Comparison of the molecular weights of identified peptides for different block ages for the HILIC method | 1. Kolmogorov-Smirnov test: D = 0.085, p < 0.01  2. Kruskal–Wallis test:  H(2) = 9.57, p = 0.0084 | 1. p < 0.05 therefore the distribution is significantly different from a normal distribution, i.e. it is non-normal.  2. There is a significant difference (p < 0.05) between the molecular weights of peptides generated from 1, 5 and 10-year old blocks when using the HILIC method. |
| Figure 6 C: Comparison of the isoelectric points of identified peptides for different protein purification methods for 1-year-old blocks | 1. Kolmogorov-Smirnov test: D = 0.17, p < 0.01  2. Kruskal–Wallis test:  H(2) = 338.16, p < 0.0001 | 1. p < 0.05 therefore the distribution is significantly different from a normal distribution, i.e. it is non-normal.  2. There is a significant difference (p < 0.05) between the isoelectric points of peptides generated via the different protein purification/sample preparation methods for 1-year old blocks. |
| Figure 6 C: Comparison of the isoelectric points of identified peptides for different protein purification methods for 5-year-old blocks | 1. Kolmogorov-Smirnov test: D = 0.17, p < 0.01  2. Kruskal–Wallis test:  H(2) = 774.40, p < 0.0001 | 1. p < 0.05 therefore the distribution is significantly different from a normal distribution, i.e. it is non-normal.  2. There is a significant difference (p < 0.05) between the isoelectric points of peptides generated via the different protein purification/sample preparation methods for 5-year old blocks. |
| Figure 6 C: Comparison of the isoelectric points of identified peptides for different protein purification methods for 10-year-old blocks | 1. Kolmogorov-Smirnov test: D = 0.17, p < 0.01  2. Kruskal–Wallis test:  H(2) = 374.56, p < 0.0001 | 1. p < 0.05 therefore the distribution is significantly different from a normal distribution, i.e. it is non-normal.  2. There is a significant difference (p < 0.05) between the isoelectric points of peptides generated via the different protein purification/sample preparation methods for 10-year old blocks. |
| Figure 6 C: Comparison of the isoelectric points of identified peptides for different block ages for the APFAR method | 1. Kolmogorov-Smirnov test: D = 0.17, p < 0.01  2. Kruskal–Wallis test:  H(2) = 81.01, p < 0.0001 | 1. p < 0.05 therefore the distribution is significantly different from a normal distribution, i.e. it is non-normal.  2. There is a significant difference (p < 0.05) between the isoelectric points of peptides generated from 1, 5 and 10-year old blocks when using the APFAR method. |
| Figure 6 C: Comparison of the isoelectric points of identified peptides for different block ages for the DRP method | 1. Kolmogorov-Smirnov test: D = 0.17, p < 0.01  2. Kruskal–Wallis test:  H(2) = 19.10, p < 0.0001 | 1. p < 0.05 therefore the distribution is significantly different from a normal distribution, i.e. it is non-normal.  2. There is a significant difference (p < 0.05) between the isoelectric points of peptides generated from 1, 5 and 10-year old blocks when using the DRP method. |
| Figure 6 C: Comparison of the isoelectric points of identified peptides for different block ages for the HILIC method | 1. Kolmogorov-Smirnov test: D = 0.17, p < 0.01  2. Kruskal–Wallis test:  H(2) = 40.55, p < 0.0001 | 1. p < 0.05 therefore the distribution is significantly different from a normal distribution, i.e. it is non-normal.  2. There is a significant difference (p < 0.05) between the isoelectric points of peptides generated from 1, 5 and 10-year old blocks when using the HILIC method. |
| Figure 11: Percentage of peptides containing oxidised methionine for APFAR processed samples | 1. Shapiro–Wilk test:  W = 0.920, p = 0.002  2. Kruskal–Wallis test:  H(2) = 1.23, p = 0.54 | 1. p < 0.05 therefore the distribution is significantly different from a normal distribution, i.e. it is non-normal.  2. There are no significant differences (p > 0.05) in levels of oxidised peptides between the different block ages processed via the APFAR method. |
| Figure 11: Percentage of peptides containing oxidised methionine for DRP processed samples | 1. Shapiro–Wilk test:  W = 0.944, p = 0.019  2. Kruskal–Wallis test:  H(2) = 0.86, p = 0.65 | 1. p < 0.05 therefore the distribution is significantly different from a normal distribution, i.e. it is non-normal.  2. There are no significant differences (p > 0.05) in levels of oxidised peptides between the different block ages processed via the DRP method. |
| Figure 11: Percentage of peptides containing oxidised methionine for HILIC processed samples | 1. Shapiro–Wilk test:  W = 0.860, p = < 0.0001  2. Kruskal–Wallis test:  H(2) = 3.38, p = 0.18 | 1. p < 0.05 therefore the distribution is significantly different from a normal distribution, i.e. it is non-normal.  2. There are no significant differences (p > 0.05) in levels of oxidised peptides between the different block ages processed via the HILIC method. |
